# *Portulaca oleracea* L seed extracts counteract diabetic nephropathy through SDF-1/IL10/PPARγ–mediated tuning of keap1/Nrf2 and NF-κB transcription in Sprague Dawley rats

**DOI:** 10.1186/s13098-024-01330-y

**Published:** 2024-05-30

**Authors:** Wessam M. Aziz, Samia A. Ahmed, Sylvia E. Shaker, Dalia B. Fayed, Nadia S. Metwally, Heba Shawky

**Affiliations:** Therapeutic Chemistry Department, Pharmaceutical Industries and Drug Research Institute, National Research Centre, Dokki, Cairo, 12622 Egypt

**Keywords:** Purslane, Diabetic nephropathy, Nrf2/Keap1, PPARγ, Oxidative stress, Inflammation

## Abstract

**Background & objective:**

: While oxidative stress is the key player driving diabetic nephropathy (DN), firm glycemic control remains the pillar prophylactic measure. Purslane was extensively described as a potent hypoglycemic and hypolipidemic agent owing to its rich content of antioxidants. Therefore, this report aimed to assess the renoprotective potentials of methanol (MO) and methylene chloride (MC) fixed oil extracts of purslane seeds in a diabetic nephropathy (DN) model.

**Methods:**

Purslane seeds were extracted using absolute methanol and methylene chloride, and type-1 diabetes was induced with a single 55 mg/kg dose of Streptozotocin (STZ) dissolved in 100 mmol/L citrate buffer (pH 4.5), and then diabetic animals were received MO, MC, for 42 consecutive days to compare their antidiabetic effect relative to the reference drug “Losartan”. Renal functions and DN biomarkers were weekly assessed, and the relative expression of different oxido-inflammatory mediators was quantified in diabetic kidneys by RT-PCR. Data were statistically analyzed using GraphPad Prism 9.0.2.

**Results:**

The oral administration of MO and MC extracts (250 mg/kg/day) significantly ameliorated the body weight loss (*P <* 0.0001 / each), fasting blood glucose levels (FBG) (*P* < 0.0001 / each), urine volume (*P* < 0.0001 / each), as well as serum creatinine (*P <* 0.0001 / each), uric acid (*P* = 0.0022, 0.0052), and blood urea nitrogen (BUN) (*P* = 0.0265, 0.0338); respectively, compared with the untreated diabetic rats. In addition, both extracts restored the effectuality of antioxidative machinery in diabetic kidneys as indicated by a significant reduction of ROS accumulation and lipid peroxidation; higher GSH content, and promoted activity of glutathione reductase and superoxide dismutase antioxidant enzymes (*P* < 0.0001 / each). Histologically, both extracts alleviated the DN-structural alterations including the glomerular congestion and tubular degeneration, with MC-treated kidneys showing near to normal architecture. The transcription profiles of all treated kidneys revealed a significantly downregulated expression of TNF-α, IL-6, Keap1 and NF-κB genes, concomitant with a significant upregulation of SDF-1, IL-10, Nrf2, HO-1, and PPARγ gene expression (*P* < 0.0001 / all).

**Conclusion:**

These findings highlight the remarkable DN-prophylactic potentials of purslane extracts mediated by neutralizing the hyperglycemia-induced ROS accumulation, and circumventing the downstream inflammatory cascades, surpassing the reference angiotensin receptor blocker; i.e. Losartan.

**Supplementary Information:**

The online version contains supplementary material available at 10.1186/s13098-024-01330-y.

## Graphical Abstract

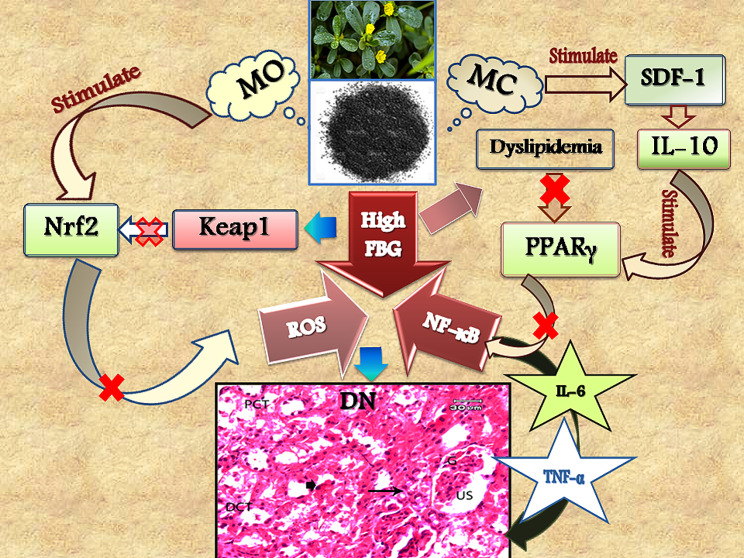

## Introduction

While the living standards improve in many countries worldwide, the number of people living with diabetes mellitus (DM) continually increases. As per the last WHO report, the global prevalence of DM is estimated at 422 million incidences and 1.5 million diabetes-related deaths per year [1]. The main pathological factor driving the long-term complications of hyperglycemia lies in the excessive accumulation of reactive oxygen radicals (ROS), which results from the deactivation of the antioxidative enzymes through glycation [2]. This typically triggers uncontrolled inflammatory responses that further potentiate ROS production, which eventually perpetuates the pathological condition [3].

One of the imperative DM complications is diabetic nephropathy (DKD) (aka. Diabetic nephropathy (DN)), in which glomerular injury and hyperflitration are directly induced by hyperglycemia [4]. As the exact mechanism underlying the onset of DN is still dialectical, halting its progression into end-stage renal disease (ESRD) remains challenging. However, protein kinase C (PKC) is reportedly the main player orchestrating the glomerular injury when activated by high glucose concentration; either through the transforming growth factor (TGF)-β-mediated overproduction of extracellular matrix (ECM) [5], or by stimulating the mitogen-activated protein kinase (MAPK) signaling pathway that promotes excessive ROS production expediting the glomerular apoptosis [6]. Recently, renal lipid deposition or lipotoxicity was proposed as another key contributor in the progression of DN, where dyslipidemia; i.e. dysregulated lipid homeostasis, potentiates the renal uptake of free fatty acids (FFA) that accumulate in plasma under diabetic conditions. The renal ω-oxidation of FFA then predominates the mitochondrial β-oxidation, which triggers renal lipotoxicity by the accumulated lipids and ROS [7]. Either way, there is a consensus that oxidative stress is the key player driving hyperglycemic nephropathy; therefore, firm glycemic control remains the pillar prophylactic measure.

Currently, two categories of kidney-protective drugs are most commonly described for diabetic patients; angiotensin-converting enzyme (ACE) inhibitors (e.g. Lisinopril, Enalapril, Benazepril), and angiotensin receptor blockers (ARBs) (e.g. Losartan, Olmesartan, Valsartan) [8]. However, the long-term usage of these drugs is associated with several side effects; some of which might be serious, which prompts a relentless pursuit of effective alternatives from natural sources. In this domain, the renoprotective role of various phytochemicals has been highlighted in several studies approaching the complications of diabetes [9–11]. Most of these agents adopt a common strategy of renoprotection; stimulating the nuclear factor-erythroid-2-related factor 2 (Nrf2) which is the key element of cellular antioxidant machinery [9–12].

In detail, Nrf2 is the master transcription factor that activates the expression of phase II detoxifying enzymes, including heme oxygenase-1 (HO-1), superoxide dismutase (SOD), glutamyl cysteine synthetase (GCLC), and γ-glutamylcysteine synthetase (γ-GCS) [12]. In addition, Nrf2 restrains cellular inflammation and fibrosis by suppressing the nuclear factor kappa (NF-κB) and transforming growth factor (TGF)-β; respectively [13]. Under physiological conditions, the cytoplasmic Nrf2 is typically bound with the ROS-sensor Kelch-like ECH-associated protein (Keap)-1, which maintains the cytoplasmic Nrf2 levels by ubiquitinated proteasomal degradation. Upon stimulation by ROS, the Nrf2 protein releases and translocates into the nucleus to recruit the transcription of different antioxidant enzymes that counteract the cellular oxidative damage [14]. Notably, the Keap1 transcription is significantly upregulated in diabetic kidneys, while that of Nrf2 as well as the cytoplasmic levels is typically downregulated [15]. On the other hand, some phytochemicals assume renoprotection by activating the peroxisome-proliferator activated receptor (PPAR)-γ [16]; the major nuclear receptor controlling the renal lipid metabolism and energy homeostasis, and reportedly implicated in several renal disorders [17].

In the same line, the wide-span bioactivities of the succulent weed *Portulaca oleracea* L. (Purslane) have been extensively described in the folk medicine domain, where several reports highlighted its hypoglycemic, hypolipidemic, potent antioxidant and anti-inflammatory potentials among others [18–20]. Besides the rich content of ω-3 fatty acids, purslane exhibits potent hypocholesterolemic and hypolipidemic activities owing to their high content of betalain pigments [18, 21]. In our previous study, methanol (MO) and methylene chloride (MC) purslane seed extracts demonstrated remarkable antioxidant/anti-inflammatory potentials in vitro [22]. The chromatographic profiling revealed that protocatechuic acid (57.1%) represents the prominent phytoconstituent of MO extract, while ω-polysaturated fatty acids constituted ∼ 70% of the fixed oil (MC); prominently linoleic acid (43.2%) and palmitic acid (24.49%), with marginal fractions of phytosterols.

It was postulated that each extract undergoes a different anti-inflammatory mechanism that ultimately correlates with interrupting NF-κB transcription or phosphorylation/translocation. Specifically, the MO treatment of LPS-sensitized RAW 264.7 cells produced a significant inhibition of IL-1β and TNF-α production, which inferred a potential interception of NF-κB activation and subsequent nuclear translocation through neutralizing ROS trigger, whereas the fixed MC oil upregulated the levels of the anti-inflammatory cytokine IL-10, which intercepted the inflammatory cascade as indicated by the strong inverse correlation observed between IL-10 levels and the relative expression of PGE2 gene [22]. Herein, we aimed to assess these antioxidative/anti-inflammatory potentials in vivo in the context of type-1 diabetic nephropathy model, and to further explore the molecular mechanisms and signaling pathways underlying their anticipated renoprotective potentials.

## Materials & methods

### Preparation of purslane seed extracts

Methanol (MO) extract and methylene chloride (MC) fixed oil were prepared from purslane seeds according to our previous report [22]. Purslane seeds were purchased from the local markets in Cairo-Egypt and were then authenticated by the senior botanist of *El-Orman* Garden (Giza, Egypt). Seeds were thoroughly ground to obtain a fine powder, and the extracts were prepared from 1 kg of ground purslane seeds by Soxhlet apparatus using absolute methanol (MO) or methylene chloride (MC) (Sigma Aldrich, USA) at ambient temperature. The extracts were then filtered and concentrated under vacuum conditions on a rotary evaporator at 40 °C, and then stored at -20 °C for further use.

### Animals

Male Sprague Dawley rats (8–10 weeks old, 225–250 gm) were obtained from the animal house at the National Research Centre, Cairo-Egypt. Before experiments, animals were acclimated for one week at 25 °C with a 12 h /12 h darkness photo-period, with free access to standard chow and water *ad libitum*. The number of animals and their mean body weights were determined according to the targeted study. For the toxicology study, 72 rats with an average body weight of 140 ± 2.0 gm were used; while 50 other rats with a body weight range of 225–250 gm were used for DN model establishment and subsequent antidiabetic/ renoprotective assessment of purslane extracts.

### Toxicology studies

For acute toxicity assessments, 42 rats were allocated into seven weight-matched groups (*n* = 6/group) designated as sham (untreated control); high-, medium-, and low-dose groups in which animals orally received a single 5, 2.5, and 1 gm/kg doses of each extract in phosphate-buffered saline (PBS); respectively, while sham rats received the same dose volume of PBS. Animal mortality and signs of toxicity including weight change, and behavioral changes (excessive grooming, repetitive circling, somatomotor activity, eyes, mucous membranes, presence /absence of alopecia) were monitored twice daily for 14 days, and the LD_50_ value was calculated using the regression equation according to **Lorke** [23]. According to the obtained LD_50_, 1/20 of the LD_50_ was selected as the maximum concentration for repeated dosing of 20 rats (*n* = 10/group), considering the relatively long experimental period (42 consecutive days) in which animals were monitored compared to the untreated (*n* = 10) as aforementioned. The changes in body weights in each group, as well as the rate of food/water intake, were weekly recorded.

### Induction of diabetes mellitus

Type1-diabetes mellitus (T1DM) was induced in 12 h-fasting rats with intraperitoneal (i.p) administration of a single 55 mg/kg dose of Streptozotocin (STZ) dissolved in 100 mmol/L citrate buffer (pH 4.5) [24], while sham group (*n* = 10) received only the vehicle. To limit the early animal mortality associated with the sudden insulin release from the STZ-damaged β-islets; all animals were immediately injected (i.p.) with 5 µg/kg glucagon according to **Dirnena-Fusini et al.** [25]. Levels of fasting blood glucose (FBG) were estimated 3 and 7 days post-STZ treatment, and animals were considered diabetic when their FBG level was ≥ 15.5 mmol/L. The diabetic animals were allocated into four groups (*n* = 10/group) designated as:

- **DN group (untreated control)**: diabetic animals treated with an equivalent oral dose of normal saline for 42 consecutive days.
- **MO-treated group**: diabetic animals daily treated with methanol extract of purslane seeds for 42 consecutive days with a pre-determined dose according to the toxicology studies.
- **MC-treated group**: diabetic animals daily treated with methylene chloride extract of purslane seeds for 42 consecutive days with a pre-determined dose according to the toxicology studies.
- **Reference drug-treated group**: diabetic animals daily treated with 2 mg/kg of Losartan for 42 consecutive days [26].

During the experiment period of both studies, animal groups were monitored twice/day by a blinded veterinarian. Abnormal symptoms including discomfort, fatigue, pain, skin/fur erythema, or unusual eye secretions were observed, besides the animal mortality (if any) was recorded. Behavioral abnormalities including ataxia, depression or over-excitation, and altered locomotor activity were also focused. Pre-treatment animal mortality was compensated with the same number of other rats that passed the same T1DM induction protocol.

### Sampling

For toxicological assessments, blood samples were collected from the tail mid-vein and then the animals were euthanized by intraperitoneal (i.p.) administration of 100 mg/kg pentobarbital. Organs including the brain, lungs, heart, liver, kidneys, spleen, and testes were weighed and visually examined for macroscopic alterations. In the DN model, diabetic rats of each group were individually housed in metabolic cages for 24 h-urine collection. Blood samples were also weekly collected from the tail mid-vein for monitoring biochemical and inflammatory markers. Urine/blood samples were centrifuged for 10 min at 3000×*g* to obtain sera and clear urine samples for subsequent analyses. Animal euthanasia was carried out as aforementioned, and kidneys were dissected and rinsed with cold isotonic saline and then weighed to determine kidney hypertrophy index (Ki) by dividing the kidney wet weight by the body weight of each animal [27]. Sections of the collected kidneys were homogenized for the assessment of redox status.

### Assessment of biochemical and inflammatory markers

Biochemical assessments including Serum ALT, AST, total cholesterol, and triglycerides besides the renal function markers listed below were estimated in control and MO/MC-treated animals were carried out using **Biodiagnostic (Cairo, Egypt)** kits according to the user manual. The progressive hyperglycemia was monitored in diabetic rats on days 7, 14, 21, 28, 35, and 42th using a glucose oxidase-based commercial glucometer **(Accu-Chek Active, Roche Diagnostic)**. Renal functions were also monitored in terms of urine volume; blood urea nitrogen; serum/urinary creatinine, albumin, uric acid, and proteinuria at the same time points. The estimated glomerular filtration rate (eGFR) was calculated according to **Besseling et al.** [28] in individual animals using one of the following equations depending on serum creatinine levels:

Serum creatinine < 52 µmol/L: eGFR (µl/min) = 880 ×weight ^(0.695)^ × serum creatinine ^(−0.66)^ × blood urea ^(−0.391)^.

Serum creatinine > 52 µmol/L: eGFR (µl/min) = 5862×weight ^(0.695)^ × serum creatinine ^(−1.15)^ × blood urea ^(−0.391)^.

The onset and progression of DKD were monitored in diabetic rats using our previously proposed biomarkers including serum and/or urinary transferrin, fibronectin, IL-6, TNF-α, high sensitivity CRP (hs-CRP), and SDF-1; each was estimated using Instant Rat ELISA kits of **SunLong Co., Ltd (China)** following the user manual. Urinary levels of SDF-1 were normalized to concurrent urinary creatinine concentrations at each time point. The absorbance was measured at 450 nm against 630 nm (reference wavelength), and the cut-off value was determined according to the minimum detection limit of each kit. The renal redox status was evaluated in terms of tissue levels of reduced glutathione (GSH); lipid peroxidation (MDA), and the activity of glutathione reductase (GR) and superoxide dismutase (SOD) were biochemically assessed using **Biodiagnostic (Cairo, Egypt)** kits, while the of total reactive oxygen species (ROS) was estimated using Rat reactive oxygen species (ROS) ELISA kit **(SunLong Co., Ltd- China)** according to the user manual.

### Quantitative PCR

The gene expression of NF-κB, IL-6, TNF-α, IL-10, Nrf2, Keap1, HO-1, and PPARγ was evaluated in diabetic kidneys using quantitative PCR (qPCR). Total RNA was extracted from frozen renal sections preserved in RNAlater **(ThermoFisher Scientific, USA)** using PureLink™ RNA Mini Kit **(Invitrogen, USA)** according to the user manual. The complementary DNA (cDNA) was synthesized from 1 µg of RNA template using iScript™ One-Step RT-PCR Kit with SYBR®Green **(Bio-Rad, USA)** according to the manufacturer’s protocol, and then used as a template to amplify genes using the specific primer sets listed in Supplementary Table (1) along with the β-actin housekeeping gene. The cycling conditions included 95˚C for 10 s; annealing at (55–60℃) for 30 s; extension at 72˚C for 1 min, and final extension at 72˚C for 10 min; for 40 cycles. The melting curves were analyzed to confirm the presence of specific amplification and the absence of primer dimers. Data were analyzed by the comparative threshold cycle (ΔΔCt) method, and normalization was performed using the geometric mean of the β-actin gene.

### Histopathological assessments

Sections of the collected kidneys were fixed in 10% PBS-formalin. After 24 h of formalin fixation, specimens were washed and dehydrated in serial dilutions of ethanol, cleared in xylene, and finally embedded in paraffin. Paraffin blocks were sectioned at 4 μm, and the obtained sections were stained with hematoxylin and eosin (H&E) according to **Suvarna and Layton** [29]. A quantitative score was assigned for the severity of renal injury, where 0 = no histopathological change, 1 = slight (minimal, < 25%), 2 = mild (25-50%), 3 = moderate (> 50%), and 4 = severe (> 75%).

### Statistical analysis

The sample size was calculated using G-Power software version 3.1.9.7. The Priori test analysis of the five independent groups included in the study revealed a ∼ 11-fold difference of the FBG levels in the DN group relative to sham (Fig. S1.A), while it showed ∼ 12.902 and 6.408 fold difference in treated groups relative to sham **(B-D)** and DN **(E-G)** control groups; respectively, with mean difference of 9.655 and an average sample size required of three rats per group. However, ten rats were allocated to each group to achieve the effect size (f) of 9.655 and a study power of 95% (1-β error probe). A continuity-corrected squared Fishers’ exact test was used to evaluate the null hypothesis with a probability of type I error (α- error = 0.05), power = 95%. The statistical analysis of numerical data was performed using GraphPad Prism version 9.0.2 **(GraphPad, San Diego, CA)**, including the unpaired parametric t-test and/or one-way analysis of variance (ANOVA) followed by Tukey’s multiple comparison test. The correlation matrix was analyzed using Pearson correlation followed by linear regression tests. Data were expressed as mean ± SD in each group, and two-tailed *P*-values < 0.05 were considered statistically significant.

## Results

### Biosafety of purslane seed extracts

No animal mortality or acute signs of toxicity were observed during the 14-day experimental period, and both MO and MC extracts were safe up to 5 g/kg single dose. Therefore, a dose of 250 mg/kg was selected for repeated administration. No significant change in the organosomatic indices, or food and water intake (data not shown) was observed during or at the end of the dosing period. However, the MC-treated animals showed a moderate reduction of body weight relative to the sham animals starting from the 21st day of treatment (*P* = 0.0068–0.022), unlike the MO-treated animals that showed no significant difference along the experimental period. The repeated MO dosing produced significantly lower blood glucose levels (*P* = 0.0162), while the MC dosing exhibited a slight elevation of serum albumin (*P* = 0.0044). Both treatments exhibited a significant reduction of total cholesterol and triglycerides (*P* < 0.0001 / each), with a higher effect observed in MO-treated animals in the former (*P* < 0.0001), and a higher effect observed in MC-treated animals in the latter (*P* = 0.0003). The treated animals showed lower levels of ALT (*P* = 0.0001, 0.0033; respectively) and also AST despite the absence of statistical difference. As both extracts were investigated as renoprotective candidates, different parameters of renal functions were also assessed after repeated dosing. Results showed that both MO- and MC-treated groups had significantly lower levels of serum uric acid (*P* = 0.0001, 0.0024; respectively), and urinary creatinine (*P <* 0.0001 / each), while no significant difference was found in BUN, proteinuria, as well as creatinine clearance and eGFR rates. Meanwhile, mild elevation of serum creatinine (*P* = 0.0001, 0.0069), and urine volume (*P* = 0.0092, 0.0232) were observed in both MO- and MC-treated groups; respectively. Changes in body weight during the dosing period and the terminal values of organosomatic index and blood biochemistry parameters are listed in Supplementary Tables 2, 3, and 4; respectively.

### MO and MC treatments ameliorate hyperglycemia-associated symptoms

All the classical signs of diabetes including elevated blood glucose levels accompanied by weight loss, polyphagia, polydipsia, and polyuria were significantly pronounced in the untreated diabetic animals in the DN control group. The sham animal group had steady FBG levels and a normal weight gain pattern consistent with food consumption rate along the study period, whereas untreated diabetic animals in the DN group showed significantly higher FBG levels and water intake/ food consumption rates with lower mean body weight in all time points compared with the sham animals (*P <* 0.0001 / each). The MO-treated diabetic rats demonstrated a rapid improvement regarding FBG levels, body weight, as well as food/water consumption after only 7 days of treatment, and maintained a steady pattern. Meanwhile, the improvement of DM-associated symptoms was observed in those receiving the MC extract and Losartan after 14 days of treatment. The untreated diabetic animals showed a 31.5% increment of terminal FBG levels, concomitant with 27.26% weight loss compared with the initial values (*P <* 0.0001 / each), while both MO and MC extracts produced 67.35 and 63.1% lower FBG levels; respectively compared with the untreated diabetic rats (*P* < 0.0001 / each) at the same time point. Compared with Losartan-treated animals, the terminal FBG levels showed no significant difference in MO-treated rats unlike those treated with MC that showed significantly higher FBG levels compared with both Losartan- and MO-treated animals (*P* < 0.0001, 0.0038; respectively), while the latter showed a significantly higher terminal body weight relative to both MC- and Losartan-treated animals (*P <* 0.0001 / each). Changes in body weight, blood glucose, and food/water intake in different animal groups are detailed in Table (1).

**Table 1:** Changes in body weight, blood glucose, and food/water intake in untreated and treated diabetic rats in different groups

Parameter	Group	Days					
		**7**	**14**	**21**	**28**	**35**	**42**
FBG Change % (mmol/L)	***Sham***	1.95 ± 0.28*	1.94 ± 0.25*	1.66 ± 0.32*	1.07 ± 0.26*	0.77 ± 0.27*	0.916 ± 0.24*
	***DN***	6.85 ± 0.38*^a^	10.7 ± 0.48*^a^	11.77 ± 0.56*^a^	16.64 ± 0.64*^a^	26.86 ± 0.99*^a^	31.5 ± 0.76*^a^
	***DN + MO***	-25.59 ± 6.31^ab^	-28.84 ± 3.03^ab^	-39.82 ± 4.42^ab^	-58.47 ± 3.93^ab^	-64.58 ± 5.46^ab^	-67.35 ± 3.36^ab^
	***DN + MC***	-10.33 ± 2.55^abc^	-45.33 ± 6.34^abc^	-42.06 ± 5.91^ab^	-56.25 ± 3.59^abc^	-60.15 ± 5.4^abc^	-63.1 ± 2.11^abc^
	***DN + Drug***	-11.48 ± 2.92*^abc^	-48.27 ± 7.66^abd^	-33.01 ± 3.22^abd^	-43.13 ± 3.73^abc^	-65.69 ± 5.56^abd^	-67.07 ± 4.31^abd^
Body Weight Change% (gm)	***Sham***	6.31 ± 0.56*	14.49 ± 2.82*	20 ± 3.77*	25.07 ± 3.87*	34.85 ± 4.79*	46.14 ± 5.39*
	***DN***	-6.94 ± 0.83*^a^	-8.5 ± 1.131*^a^	-11.02 ± 2.27*^a^	-16.65 ± 3.14*^a^	-22.62 ± 3.58*^a^	-27.26 ± 3.74*^a^
	***DN + MO***	3.947 ± 1.07^b^	14.54 ± 2.35^b^	22.02 ± 2.25^b^	34.77 ± 2.91^ab^	52.43 ± 3.92^ab^	72.28 ± 4.16^ab^
	***DN + MC***	0.526 ± 0.15^ac^	7.939 ± 0.94^abc^	21.65 ± 2.91^b^	34.67 ± 2.82^ab^	48.63 ± 2.77^ab^	66.67 ± 3.43^abc^
	***DN + Drug***	2.719 ± 0.69^b^	11.06 ± 1.24^abcd^	17.52 ± 1.9^abcd^	32.42 ± 2.57^abcd^	51.9 ± 3.5^abd^	69.7 ± 4.3^abcd^
Food Consumption Change% (g/day)	***Sham***	2.49 ± 0.77*	10.4 ± 1.61*	20.57 ± 2.42*	26.22 ± 2.15*	41.84 ± 3.63*	53.72 ± 4.19*
	***DN***	9.817 ± 1.19*^a^	16.05 ± 1.3*^a^	28.4 ± 2.18*^a^	40.75 ± 3.22*^a^	53.29 ± 3.35*^a^	72.89 ± 3.25*^a^
	***DN + MO***	-7.513 ± 1.012^b^	-5.191 ± 0.887^b^	-11.22 ± 1.13^ab^	-8.6 ± 1.57^b^	-19.21 ± 1.95^ab^	-26.39 ± 1.14^ab^
	***DN + MC***	-0.474 ± 0.14^ac^	-4.814 ± 0.528^bc^	-11.49 ± 1.36^ab^	-16.28 ± 1.79^ab^	-14.06 ± 1.65^ab^	-23.91 ± 1.91^ab^
	***DN + Drug***	-0.532 ± 0.17^ac^	-7.232 ± 1.61^bcd^	-13.71 ± 1.7^abcd^	-16.19 ± 2.097^abc^	-19.49 ± 1.71^ab^	-25.05 ± 1.39^ab^
Water Intake Change % (mL/day)	***Sham***	5.24 ± 0.63*	23.64 ± 1.36*	29.6 ± 2.457*	33.48 ± 3.214*	35.08 ± 3.17*	44.68 ± 3.33*
	***DN***	10.56 ± 1.18*^a^	18.88 ± 1.948*^a^	28.56 ± 2.41*^a^	35.6 ± 4.43*^a^	46 ± 3.54*^a^	52.96 ± 3.45*^a^
	***DN + MO***	-5.664 ± 1.19^ab^	-8.398 ± 1.64^ab^	-8.961 ± 1.41^ab^	-13.94 ± 2.11^ab^	-21.12 ± 1.39^ab^	-24.07 ± 1.83^ab^
	***DN + MC***	-2.668 ± 0.73^ac^	-8.791 ± 1.16^ab^	-10.88 ± 1.38^abc^	-10.75 ± 1.25^abc^	-15.1 ± 1.32^abc^	-14.55 ± 1.21^abc^
	***DN + Drug***	-3.309 ± 1.25^ac^	-9.505 ± 1.24^abcd^	-15.5 ± 1.72^acb^	-17.03 ± 1.75^abcd^	-18.77 ± 1.04^abcd^	-19.71 ± 1.58^abd^

*Change %relative to the initial value.

^a^Significant compared with the sham group at the same time point

^b^Significant compared with DN control in the same time point

^c^Significant compared with MO-treated animals in the same time point

^d^Significant compared with MC-treated animals at the same time point

### MO and MC treatments ameliorate the altered renal functional and structural markers in diabetic animals

**Fig. 1:**
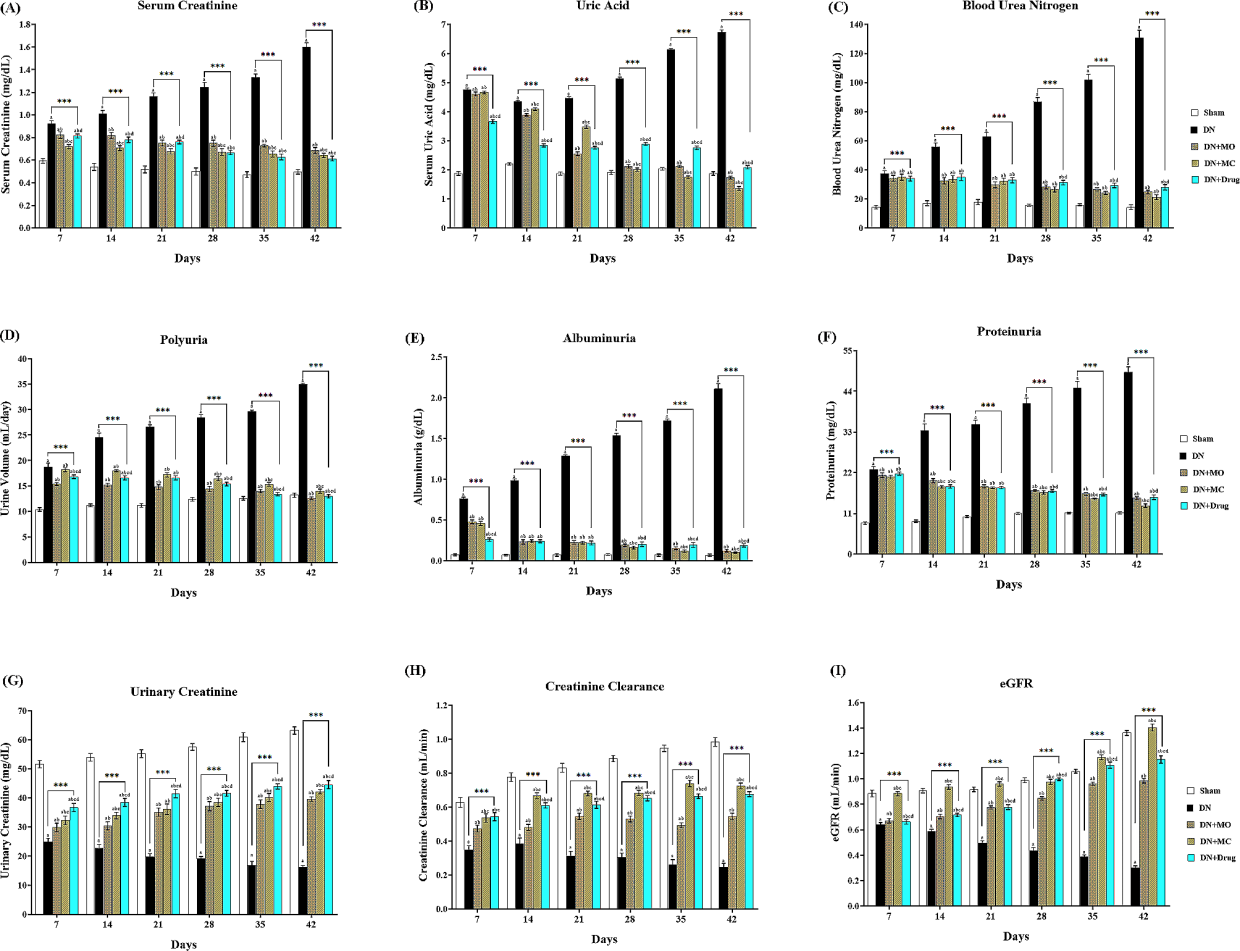
Effect of MO and MC treatments on renal injury markers. Compared with the untreated control, all treatments significantly reduced the elevated levels of serum creatinine (*P* < 0.0001 / each) **(A)**, uric acid (*P* = 0.0022 - <0.0001) **(B)**, and BUN (*P* = 0.0187–0.0338) **(C)**. The MO-, MC-, and Losartan-treated animals also showed improved urine parameters compared with the untreated animals, including a significant reduction of polyuria (*P* = 0.0402 - <0.0001) **(D)**, renal proteome (*P* = 0.0193 - <0.0001) **(E, F)**, urinary creatinine (*P* < 0.0001 / each) **(G)**, and the rates of both creatinine clearance (*P* < 0.0001 / each) **(H)** and eGFR (*P* = 0.0385 - <0.0001) **(I)** within 7 days of treatment

In line with progressive hyperglycemia, the early signs of renal injury were observed in the untreated diabetic rats between the 7th and 14th days after the STZ administration, in terms of higher serum creatinine (Fig. 1.A); uric acid (1.B), and BUN levels (1.C), in addition to significant polyuria (1.D), higher levels of albuminuria (1.E), proteinuria (1.F), and urinary creatinine (1.G) concomitant with lower creatinine clearance (1.H) and eGFR rates (1.I) compared with the sham animals (*P* < 0.0001/ each) in all time points. All treatments significantly improved the regression of renal functional and structural markers compared with the untreated animals. In detail, the MO-, MC-, and Losartan-treated animals showed lower levels of serum creatinine (*P <* 0.0001 / each), uric acid (*P* = 0.0022, 0.0052, < 0.0001; respectively), and BUN levels (*P* = 0.0265, 0.0338, 0.0187; respectively) compared with the untreated after 7 days of treatment with no significant difference observe between the three treated groups. Similarly, a significant reduction of the polyuria (*P* < 0.0001, 0.0402, < 0.0001; respectively), renal proteome including levels of albuminuria (*P <* 0.0001 / each) and proteinuria (*P* = 0.0054, 0.0003, 0.0193; respectively), and urinary creatinine (*P <* 0.0001 / each) was observed in the three treated groups after 7 days of treatment, which also extended to improved rates of creatinine clearance (*P <* 0.0001 / each) and eGFR (*P* = 0.0123, < 0.0001, 0.0385; respectively). As demonstrated in Fig. (1.D). The MO treatment showed a persistently higher effect on the polyuria in all time points recorded compared with MC and Losartan, while the MC exhibited significantly higher improvement of renal proteome and both creatinine clearance and eGFR rates relative to MO and Losartan (*P* < 0.01 / each) (1.E-I).

### MO and MC treatments halt the progression of diabetic nephropathy

**Fig. 2:**
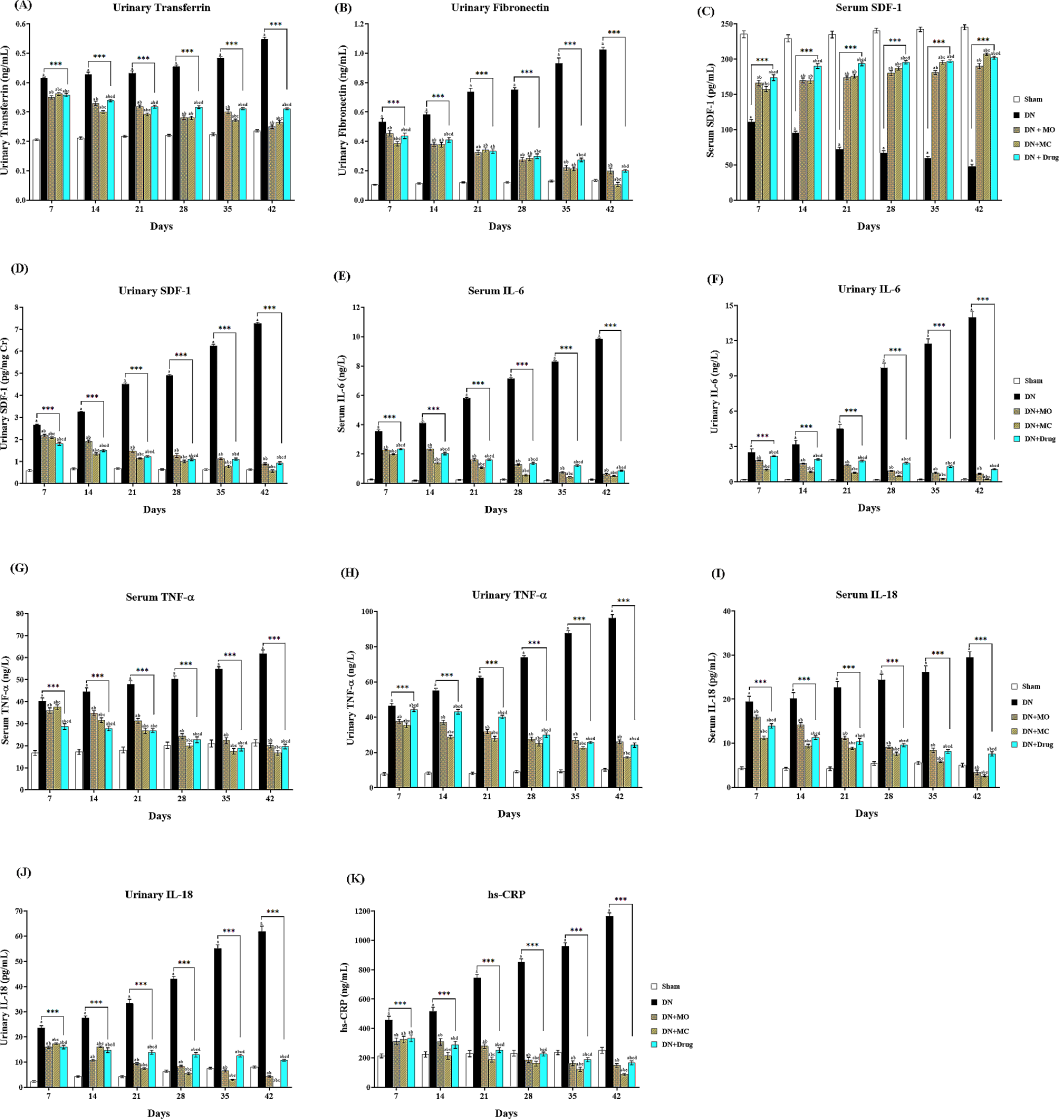
Effect of MO and MC treatments on early markers of diabetic nephropathy. All treatments halted the progression of the DN reflected by the significant reduction of urinary transferrin **(A)**, fibronectin **(B)**, urinary SDF-1 **(D)**, serum/urinary IL-6 **(E, F)**, TNF-α **(G, H)**, and IL-18 **(I, J)**, in addition to serum hs-CRP **(K)**, and the significant upregulation of serum SDF-1 levels **(C)** comparing with the untreated control (*P* < 0.0001 / each). The regulatory onset of each treatment was observed at different time points, where the MO extract showed the modulatory effect on urinary transferrin and IL-18 besides serum TNF-α and SDF-1 during the first week of treatment, while the MC extract showed higher activity on urinary SDF-1, serum/urinary IL-6, urinary TNF-α and serum IL-18 at the same time point. The ultimate values of investigated markers were almost normalized in MC-treated animals

The early markers of diabetic nephropathy were detected in the untreated diabetic animals (DN) after 14 days of STZ dosing, where the urinary levels of transferrin (Fig. 2.A); fibronectin (2.B); SDF-1 (2.D); IL-6 (2.F); TNF-α (2.H), and IL-18 (2.J) were found to be significantly higher than those in the sham and treated diabetic animals, concomitant with significant reduction of serum SDF-1 (2.C), and elevated IL-6 (2.E); TNF-α (2.G); IL-18 (2.I), and hs-CRP levels (2.K) at all time points (*P <* 0.0001 / each). The three treatments; particularly the MC extract, halted the progression of the DN as demonstrated by significant improvement of the dysregulated markers’ levels until they became near the baseline or undetectable levels by the end of the study. The onset of each treatment’s modulatory effect was observed at different time points, where the MO extract exhibited higher activity in reducing the elevated levels of urinary transferrin, urinary IL-18, and serum TNF-α by 3.31, 9.24, and 4.31% (*P* = 0.0036, 0.0003, 0.0454); respectively, while it upregulated serum SDF-1 levels by 5.27% (*P* = 0.0046) relative to MC extract after 7 days of treatment. Meanwhile, the latter exhibited superior regulatory effects on urinary SDF-1, serum/urinary IL-6, urinary TNF-α, and serum IL-18 at the same time point compared with MO extract, where treated animals had 4.3, 12.35, 45.63, 5.98, and 29.64% lower levels (*P* = 0.0091, < 0.0001, < 0.0001, 0.0109, 0.0001); respectively, while they had 9.7, 10.26, 42.16, and 43.91% lower levels of transferrin, serum TNF-α, urinary SDF-1, and hs-CRP levels (*P* < 0.0001, < 0.0001, 0.0008, < 0.0001, and 0.0001); respectively at the 14th day. Regarding fibronectin levels, no significant difference was observed in MC-treated animals relative to those treated with the MO extract before the 42th day, where the animals showed 84.83% (*P* < 0.0001) lower levels, while the MC treatment exhibited a higher inhibitory effect on urinary IL-18 levels at the 21st day of treatment, and the treated animals ended up with undetectable levels. Compared with Losartan, the MO-treated animals showed 2.29, 3.84, 19.9, and 17.75% (*P* = 0.0305, 0.0106, < 0.0001/each; respectively) lower transferrin, serum/urinary IL-6, urinary TNF-α; respectively at the 7th day of treatment, while the MC-treated animals showed 13.77, 18.47, 120.49, 25.24, and 24.04% (*P* = 0.0007, < 0.0001 / each; respectively) lower fibronectin, serum/urinary IL-6, urinary TNF-α, and serum IL-18; respectively at the same time point.

### MO and MC treatments maintain the structural integrity of diabetic kidneys

**Fig. 3:**
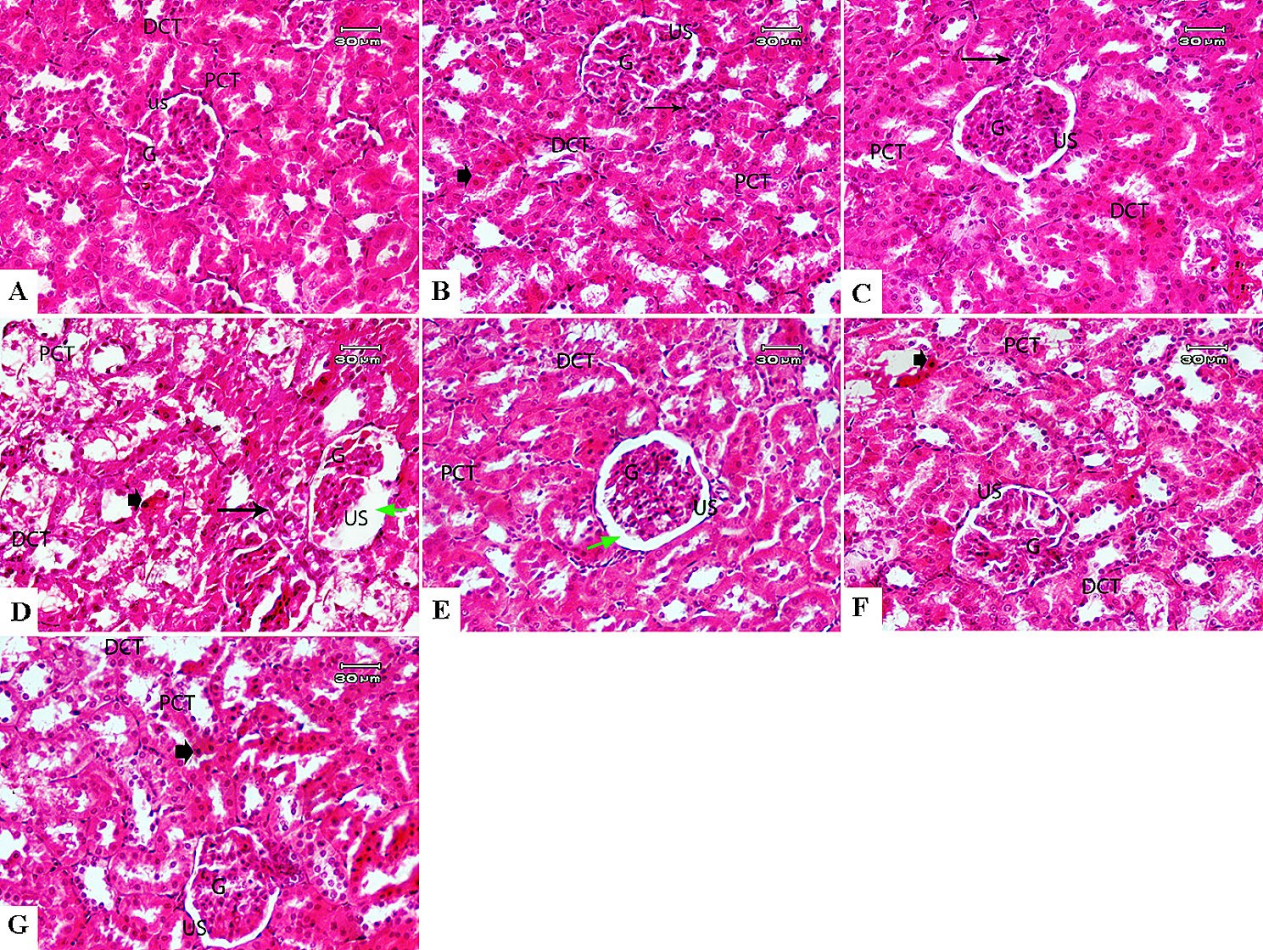
Effect of MO and MC treatments on histopathological alterations in diabetic kidneys. Sections of normal kidneys **(A)** and control groups receiving only MO **(B)** and MC **(C)** extracts showed the normal-appearing architecture of the glomeruli (G); urinary space (US), Proximal (PCT), and distal (DCT) convoluted tubules. The untreated diabetic kidneys **(D)** showed severe glomerular and tubular alterations, including collapsed glomerular tuft (G) associated with wide urinary space (US), epithelial desquamation of proximal (PCT) and distal (DCT) convoluted tubules, cellular swelling, intra-cytoplasmic vacuolization **(thin arrow)**, and pyknotic nuclei **(arrowhead)**. MO treatment has markedly alleviated the periglomerular cellular infiltration in diabetic kidneys **(E)**, therefore, the glomeruli were less congested and/or hypercellular, and merely mild degeneration was observed in proximal (PCT) and distal (DCT) convoluted tubules. The widened urinary space (US) **(thin green arrows)** was not normalized. Both MC- and Losartan-treated kidneys **(F, G)** showed normal structure of the glomerulus (G), and urinary space. (US) Proximal (PCT) and distal (DCT) convoluted tubules with few cellular infiltrations around glomeruli **(thin arrow)**

The absolute weights and hypertrophy index (Ki) of the untreated kidneys were significantly higher than those observed in the control (*P <* 0.0001 / each), while the MC extract normalized both the weight and Ki value in treated kidneys with no statistical difference observed relative to the control. Meanwhile, both MO and Losartan-treated groups showed moderately higher kidney weights (17.2, 22.44%; *P* = 0.0279, 0.0016; respectively) and Ki values (19.92, 23.62%; *P* = 0.0497, 0.0105; respectively) compared with the MC-treated despite the lower values relative to the untreated control (*P <* 0.0001 / each). The microscopic assessment of the renal sections obtained from the sham group (Fig. 3.A) revealed normal kidney architecture. Glomeruli (G) were intact with normal-sized basement membrane and Bowman’s (urinary) space, and both proximal (PCT) and distal convoluted tubules (DCT) were normal-appearing, with obvious vesicular round nuclei and granular cytoplasm. Similar findings were observed in sections obtained from control animals receiving only the MO (3.B) and MC extracts (3.C). In diabetic renal Sect.  (3.D), severe glomerular congestion associated with massive periglomerular cellular infiltrations was observed. The untreated glomeruli showed marked hypercellularity, with markedly collapsed tufts that resulted in urinary space widening. Both proximal and distal convoluted tubules showed marked degenerative changes including epithelial desquamation, necrosis, cellular swelling, intra-cytoplasmic vacuolization, and darkly stained pyknotic nuclei, with moderate hemorrhage observed in interstitial tissue. The MO treatment (3.E) alleviated the glomerular congestion and reduced the tubular degeneration compared with the untreated kidneys; however, moderate glomerular hypercellularity associated with widened urinary space (US) and mild periglomerular cellular infiltration were still detectable. Meanwhile, the MC-treated kidneys showed near to normal architecture with minute cellular infiltration, intact glomeruli with well-defined Bowman’s space, and intact convoluted tubules with mild degeneration and pyknotic nuclei (3.F). Notably, reformation of renal tubular epithelium was also observed. Similarly, the Losartan-treated kidneys showed normal architecture with few cellular infiltrations around glomeruli and mild degeneration of proximal (PCT) and distal (DCT) convoluted tubules and pyknotic nuclei (3.G). Renal injury scores based on microscopic assessments are listed in Table (2).

**Table 2:** Scoring of macro-/microscopic alterations in untreated and treated diabetic kidneys in different groups

*Renal Injury Scores	Sham	MO	MC	DN	DN + MO	DN + MC	DN + Drug
Kidney Weight (gm)	0.69 ± 0.03	0.67 ± 0.05	0.69 ± 0.04	1.21 ± 0.04^a^	0.86 ± 0.05^ab^	0.71 ± 0.03^bc^	0.92 ± 0.06^abd^
Ki (%)	0.21 ± 0.019	0.22 ± 0.01	0.20 ± 0.02	0.68 ± 0.024^a^	0.29 ± 0.015^ab^	0.23 ± 0.022^bc^	0.3 ± 0.018^abd^
Glomerular Hypertrophy	0/4	0/4	0/4	4/4	2/4	0/4	0/4
Glomerular Congestion	0/4	0/4	0/4	4/4	2/4	0/4	1/4
Thickened base membrane	0/4	0/4	0/4	3/4	1/4	0/4	0/4
Tubular Degeneration	0/4	0/4	0/4	4/4	2/4	1/4	1/4
Tubular Vacuolation	0/4	0/4	0/4	3/4	2/4	1/4	1/4
Cellular Infiltration	0/4	0/4	0/4	4/4	1/4	1/4	2/4
Widened Urinary Space	0/4	1/4	0/4	4/4	1/4	0/4	0/4

^a^Significant compared with the sham group

^b^Significant compared with DN control

^c^Significant compared with MO + DN animals

^d^Significant compared with MC + DN animals

*Renal Injury Score: 1 = Slight/minimal (> 25%); 2 = Mild (25–50%); 3 = Moderate (50–75%), 4 = Severe (> 75%)

### MO and MC treatments restore the effectuality of the antioxidant system in diabetic kidneys

**Fig. 4:**
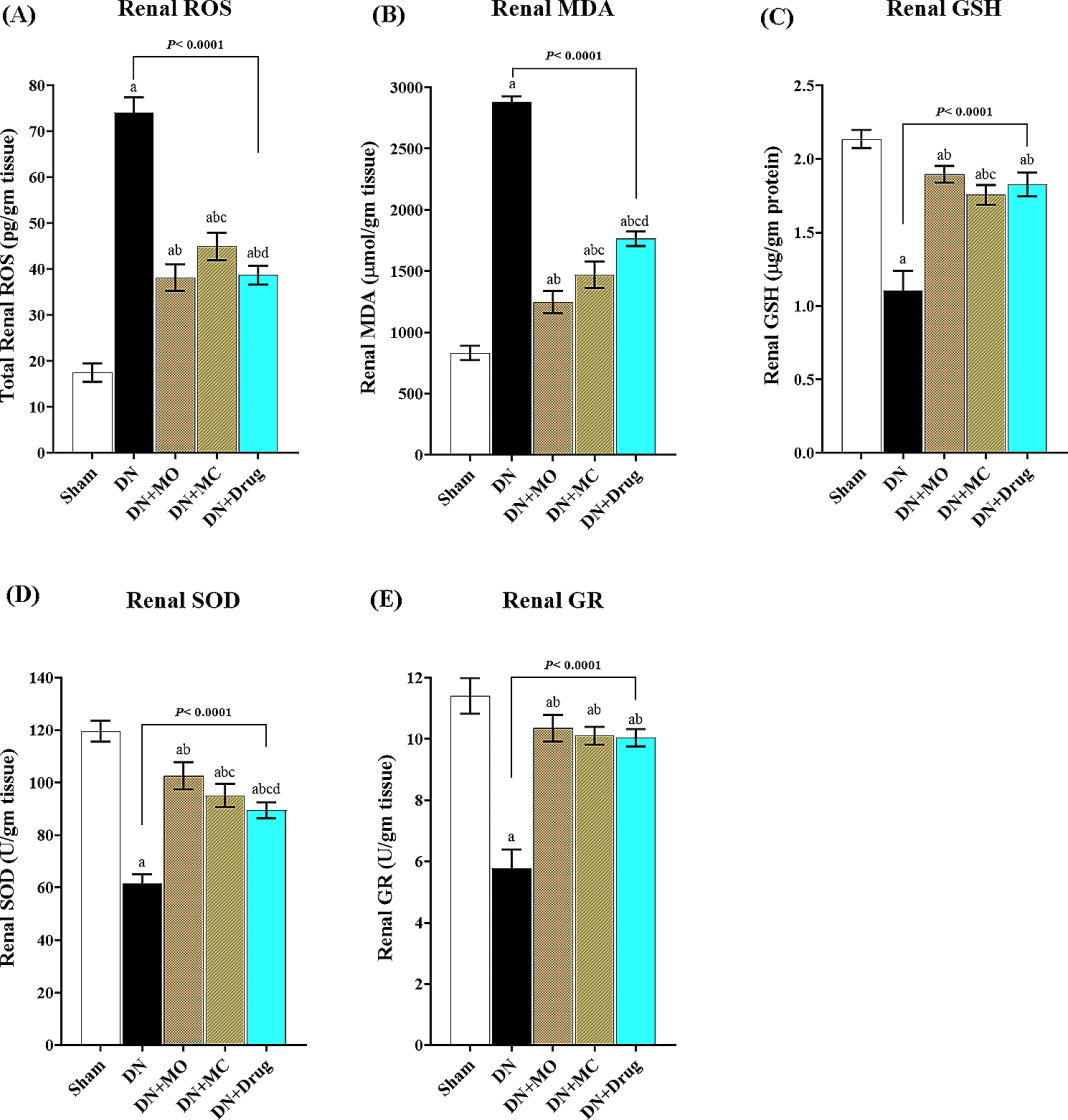
Effect of MO and MC treatments on redox status in diabetic kidneys. Significant elevation of total ROS **(A)** and MDA production **(B)** was observed in the diabetic kidneys, concomitant with significant reduction of reduced GSH **(C)**, and lower activity of the antioxidant enzymes showed glutathione reductase (GR) and superoxide dismutase (SOD) **(D, E)** when compared with normal kidneys (*P* < 0.0001 / each). The three treatments significantly suppressed the ROS accumulation and lipid peroxidation, while they significantly elevated the tissue levels of GSH, and promoted the activity of glutathione reductase (GR) and superoxide dismutase (SOD) in diabetic kidneys (*P* < 0.0001 / each). Notably, the highest improvement of distorted renal redox was observed in MO-treated kidneys

The accumulation of reactive oxygen associated with excessive lipid peroxidation was more pronounced in the untreated diabetic kidneys. Compared with sham animals, kidneys of untreated diabetic animals had 4.23 and 3.45 fold higher levels of total ROS and MDA; respectively (Fig. 4.A, B), concomitant with 51.7% reduction of GSH levels (4.C) and 50.6 and 51.57% lower activity of glutathione reductase (GR) and superoxide dismutase (SOD) (4.D, E) (*P <* 0.0001 / each). The three treatments exerted significant improvement of the dysregulated antioxidant markers with the MO treatment showing the highest ameliorative effect. The MO, MC, and Losartan-treated kidneys showed 48.41, 39.33, and 47.76% reduction of total ROS, and 56.62, 48.9, and 38.65% reduction of MDA levels; respectively relative to the untreated kidneys (*P <* 0.0001 / each). Also, the three treatments upregulated the tissue levels of GSH by 71.74, 59.05, and 65.4%; respectively, and promoted the activities of both GR and SOD by 79.25, 66.42%; 75.1, 54.22%, and 73.88, 45.24%; respectively, relative to the untreated diabetic kidneys (*P <* 0.0001 / each). Compared to Losartan-treated kidneys, no statistical difference was observed in MO-treated kidneys regarding the ROS, GSH levels, or GR activity, while showing 29.29% lower MDA (*P* < 0.0001) and 12.73% higher SOD activity (*P* = 0.0008). Meanwhile, the MC-treated kidneys showed 13.9% higher ROS (*P* = 0.006) and 16.71% lower MDA levels (*P* = 0.0004), but no statistical difference was observed regarding GSH levels, GR and SOD activities.

### MO and MC treatments modulate the transcription of the oxido-inflammatory mediators in diabetic kidneys

**Fig. 5:**
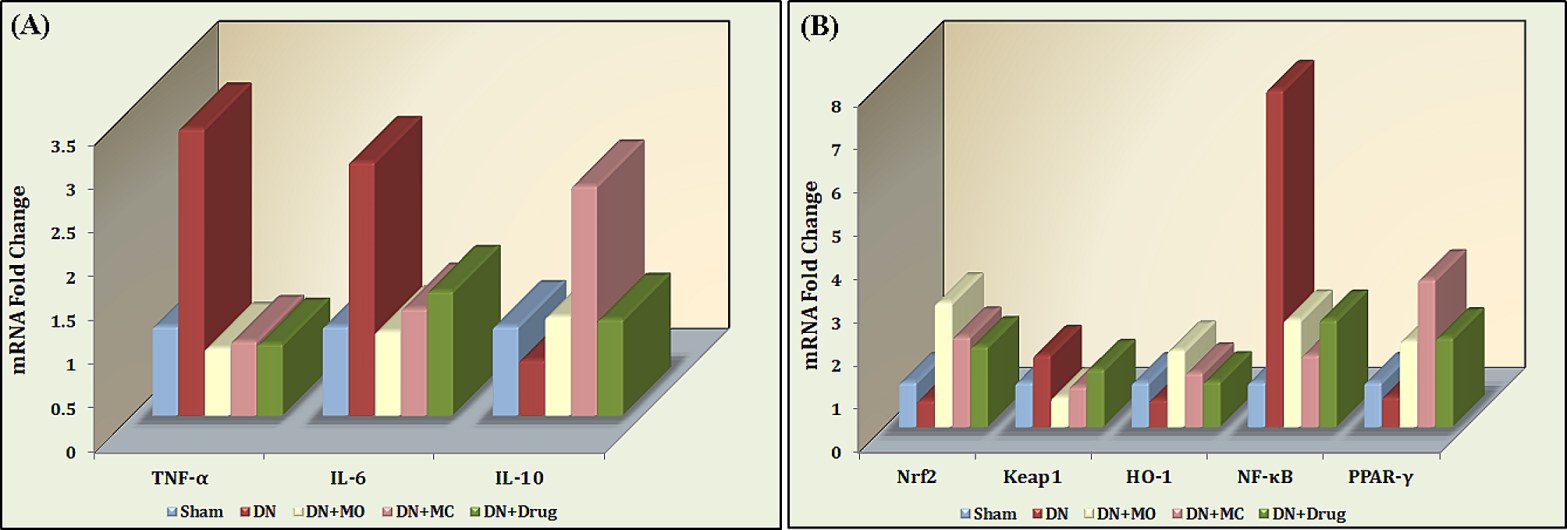
Effect of MO and MC treatments on the gene expression of oxido-inflammatory mediators in diabetic kidneys. Compared with normal kidneys, the diabetic kidneys showed 3.25, 2.87, 1.625, and 7.738 fold higher expression of TNF-α, IL-6, Keap1, and NF-κB genes, while they had 51.5, 58.5, 60 and 66.7% lower IL-10, Nrf2, HO-1, and PPARγ expression (*P* = 0.0034 - <0.0001). Both MO and MC extracts as well as Losartan treatments significantly downregulated the expression of TNF-α, IL-6, Keap1, and NF-κB genes, while upregulating the transcription of IL-10, Nrf2, HO-1, and PPARγ (*P* = 0.0004 - <0.0001). The highest expression levels of the Nrf2/HO-1 axis were observed in MO-treated kidneys, while the highest expression of PPARγ was observed in those treated with the MC extract

Relative gene expression of different mediators of the oxido-inflammatory axis including NF-κB, IL-6, TNF-α, IL-10, Nrf2, Keap1, HO-1, and PPARγ was evaluated in diabetic kidneys using quantitative PCR (qPCR). Results revealed a significant upregulation of TNF-α and IL-6 expression by 3.25, 2.87 fold in the diabetic kidneys (*P <* 0.0001 / each), while the expression of IL-10 was significantly inhibited by 51.5% (*P* = 0.0034) compared with normal kidneys (Fig. 5.A). Associated with these events, the untreated kidneys showed 58.5, 60 and 66.7% reduction of Nrf2, HO-1, and PPARγ mRNA folds (*P* < 0.0001, < 0.0001, 0.0003); respectively, while the relative expression of Keap1 and NF-κB was upregulated by 1.625 and 7.738 fold (*P <* 0.0001 / each); respectively (5.B). The relative expression of TNF-α and IL-6 genes were significantly downregulated in MO-; MC-, and Losartan-treated kidneys by 76.92, 67.2%; 74.46, 58.18%, and 75.38, 51.22%; respectively (*P <* 0.0001 / each) compared with the untreated control. Meanwhile, all treated kidneys showed significant upregulation of IL-10 expression by 53.6, 80.2, and 52.34% (*P* = 0.0002, < 0.0001, 0.0004); respectively. Concomitantly, the expression of Nrf2, HO-1, and PPARγ were significantly upregulated by 79.37, 65.32, 65.8%; 71.46, 50.82, 80.2%, and 71.35, 41.18, 67.14%in MO-, MC-, and Losartan- treated kidneys (*P <* 0.0001 / each); respectively, whereas the expression of Keap1 and NF-κB was significantly inhibited by 60, 68.44% (*P <* 0.0001 / each); 44.61, 78.91% (*P <* 0.0001 / each), and 20, 67.7% (*P* = 0.0004, < 0.0001); respectively. No significant difference in TNF-α expression level in Losartan-treated kidneys compared with both MO- and MC-treated kidneys while showing 21.2, 63.87%; 38.32, 61.31%, and 50, 30.77% higher COX2, PGE2, and Keap1 expression levels (*P* < 0.0001 / all), and 35.5, 10.78%; 41.04, 16.39% lower Nrf2 (*P* < 0.0001, 0.0189) and HO-1 (*P* < 0.0001, 0.0357); respectively. Unlike the MC-treated kidneys, the MO-treated showed 32.85% lower IL-6 expression (*P* = 0.0037) compared with those treated with Losartan, while no significant difference was detected regarding the relative expression of IL-10, NF-κB, and PPAR-γ. The MC-treated kidneys; however, showed 58.42 and 39.76% higher IL-10 and PPAR-γ expression levels, 33.17% lower NF-κB (*P* < 0.0001 / all) compared with the Losartan-treated, with no difference observed in the MO-treated.

To elucidate the signaling cascades potentially implicated in the renoprotective effect of the two purslane extracts, the correlation pattern between hyperglycemia and associated oxidative stress with the expression levels of different oxido-inflammatory mediators in diabetic kidneys was analyzed. Results revealed strong positive correlations between both FBG and ROS levels with the mRNA folds of TNF-α, IL-6, Keap1, and NF-κB, while they were negatively correlated with those of Nrf2, HO-1, PPARγ, and IL-10 (Supplementary Table 5). In contrast, the serum level of SDF-1 showed an inverted correlation pattern with the same parameters; i.e. positively correlated with Nrf2, HO-1, PPARγ, and IL-10, while negatively correlated with TNF-α, IL-6, Keap1, and NF-κB mRNA folds. On the other hand, the expression level of Nrf2 was found to be positively correlated with that of HO-1, PPARγ, and to a lower extent with IL-10, while negatively correlated with TNF-α, IL-6, and Keap 1. Also, the expression profile of PPARγ showed a strong positive correlation with that of IL-10 while being negatively correlated with both TNF-α and IL-6, unlike the NF-κB which displayed an inverted correlation pattern with the same parameters.

## Discussion

In this report, we aimed to assess the renoprotective capacity of MO and MC extracts in the type-1 DN model after a six-week treatment protocol. Assuming that they are “natural and safe”; most studies approaching natural products or plant extracts focus on their different biological activities rather than their prospected toxicology. Therefore, both extracts were first profiled for potential adverse reactions or side effects upon the relatively long administration. The toxicological assessments revealed the overall biosafety of both extracts up to 5gm/kg dose, where no signs of acute toxicity or animal mortality/morbidity were observed during the 14-day-assessment period. In addition, the repeated dosing of both extracts showed no adverse effect on the organosomatic indices or food/water consumption. Unlike the MO-treated animals, a minimal percentage of weight reduction (1.38%) was observed in the MC-treated animals relative to the untreated control, which can be attributed to the anti-obesity activity of purslane seeds fixed oils associated with their potent hypolipidemic activity; particularly observed in diabetic animals [19, 20].

Treated animals also showed a general improvement in blood biochemistry, including lower values of total cholesterol, triglycerides, liver transaminases, blood urea nitrogen, and uric acid, besides the blood glucose despite the absence of statistical difference in the MC-treated animals. A moderate elevation of urine volume was also observed in all treated animals; particularly the MO-treated, which might be attributable to the reported antidiabetic activity of purslane seeds [19, 30]. Meanwhile, the altered levels of serum albumin and serum/urinary creatinine lay within the normal range reported for SD rats [31], where the latter neither affected the creatinine clearance or eGFR rates, nor the proteinuria levels. Based on these findings, a dose of 250 mg/kg (1/20 of the LD_50_ calculated) of each extract was selected for renoprotective assessments in diabetic rats.

In contrast to our previous report [32], a single STZ dose was sufficient to establish a persistent hyperglycemia in Sprague Dawley (SD) rats, which was manifested by the excessive polyuria, accelerated rate of weight loss, and higher consumption of food and water among other classical signs of diabetes mellitus observed one week after the STZ induction. Worthy mentioning, the assessment of renal injury/nephropathy markers was initiated two weeks after the induction of DM, to bypass the transient nephrotoxic effect of STZ and guarantee that the induced renal injury is hyperglycemia-associated [33]. The early signs of renal nephropathy were pronounced in the untreated diabetic animals after two weeks of DM induction and maintained an escalating pattern in line with progressive hyperglycemia. Of note, the dynamics of renal injury observed herein were completely different from that previously reported by **Ahmed et al.** [32], which can be expected given the higher susceptibility of SD rats to the STZ diabetogenic effect attributed to their inherent hypertensive nature [34].

As might be expected, the dysregulated redox was more pronounced in diabetic kidneys as indicated by ROS accumulation, lipid hyper-peroxidation, concomitant with lower GSH content, and suppression of the antioxidant enzymes’ activity. These events were further reflected in the renal pathophysiology in terms of marked hypertrophy, glomerulosclerosis, interstitial bleeding, and tubular atrophy. On the molecular level, the FBS/ROS axis was strongly implicated in the NF-κB-mediated overexpression of proinflammatory cytokines (TNF-α, IL-6); suppression of the antioxidant Nrf2/HO-1 axis through the upregulated expression of the Nrf2-repressor Keap1 [13, 35], in addition to the downregulated expression of IL-10 and the nuclear receptor PPARγ responsible for the homeostasis of inflammatory responses [17, 36].

Both purslane seed extracts as well as Losartan (reference drug), significantly ameliorated the hyperglycemic-associated symptoms, where all treated animals showed > 60% lower FBG levels and 40% higher body weight compared with the untreated animals, and despite the significant difference observed relative to the non-diabetic animals. Still, the MO extract showed a rapid onset and longer duration of the antidiabetic effect, where it significantly alleviated the hyperglycemia, weight loss, and the rate of food/water intake within one week of treatment, unlike the MC fixed oil and the Losartan that exhibited their activity after 14 days. The superior antidiabetic activity of MO extract is probably related to a higher antioxidant capacity attributed to its protocatechuic acid-rich content [22]; a potent radical scavenger that was recently highlighted as Nrf2-inducer [37]. In a similar context, both extracts significantly improved the deregulated renal functions and restored the unbalanced redox system in diabetic kidneys. Such improvement was extended to the structural integrity of treated kidneys that was almost normalized; particularly those treated with the MC fixed oil. However, each extract demonstrated a different pattern of renoprotection, where the MO extract– consistent with its anti-diabetic effect– showed a rapid onset of effect improving the early markers of renal nephropathy; however, for a short duration of action relative to the MC fixed oil that showed a better activity with a longer duration of action. These findings were not surprising given the potent anti-inflammatory activity of the ω-fatty acids-rich MC fixed oil that exhibited potent IL-10-mediated inhibition of PGE2 and COX2 transcription in RAW 264.7 macrophage cells [22].

It was crucial; though, to explore the molecular mechanism underlying the prophylactic activity demonstrated by both extracts, by assessing the effect of the diabetic milieu on the renal expression of different oxido-inflammatory mediators. Results of quantitative PCR showed a relevant pattern with the previous results, where all treatments differentially inhibited the transcription of IL-6, TNF-α, and NF-κB proinflammatory mediators, upregulated the anti-inflammatory mediators IL-10 and the nuclear receptor PPARγ, as well as the Nrf2/HO-1 detoxification axis while reducing the expression of Keap1. Noteworthy, the MO-treated diabetic kidneys showed 27.7 and 29.48% higher expression levels of Nrf2 and HO-1 genes; respectively, and 28% lower expression levels of Keap1 relative to those treated with the MC oil. Meanwhile, the latter showed 57.3% higher expression levels of PPARγ, which might be attributed to the high content of conjugated linoleic acid (CLA); one of the natural ligands that induce the key lipid sensor PPARγ transcription [38]. The implication of such differential expression in the signaling pathways underlying the prophylactic effect of each extract was further elucidated by the correlation pattern revealing the crosstalk between the FBG/ROS axis and the expression profile of those genes in diabetic kidneys.

The implication of such differential expression in the signaling pathways underlying the prophylactic effect of each extract was further elucidated by the correlation analysis. Results suggested that the renoprotective effect of MO extract is primarily mediated by modulating the Nrf2/HO-1/Keap1 transcription/pathway. This assumption was supported by the lower expression levels of Keap1 observed in MO-treated kidneys, which might have activated the nuclear translocation of stabilized NRF2 as indicated by the upregulated nuclear expression of both Nrf2 and its downstream HO-1 gene encoding the phase II detoxifying enzyme heme-oxygenase-1 [13, 35]. Furthermore, the overexpression of Nrf2 was strongly associated with the suppression of the NF-κB gene, which further extended to the IL-6 and TNF-α -encoding genes induced by phosphorylated NF-κB as indicated by the strong inverse correlation between their expression and that of Nrf2.

Altogether, these findings suggested an Nrf2-mediated inhibition of NF-κB [39, 40], which ultimately prevents the renal oxido-inflammatory injury triggered by hyperglycemia. On the other side, the correlation analysis implied that the MC fixed oil might display renoprotection through IL-10/PPARγ-mediated inhibition of the NF-κB signaling pathway. This conclusion was based on the higher expression of IL-10 observed in MC-treated kidneys, the strong positive correlation found between its transcription levels with that of PPARγ on one hand, and the strong negative correlation between their transcription levels and that of NF-κB/TNF-α/ IL-6 genes in the other. These results comply with the findings of **Scirpo et al.** [36] who reported that overexpressed PPARγ precludes the NF-κB-mediated inflammatory responses.

While the SDF-1 pleiotropic chemokine can perpetuate renal inflammation by activating the infiltration of CXCR4^+^ immune cells into the renal tissues [41], the levels of circulating SDF-1 herein were positively correlated with the expression of the renoprotective Nrf2, HO-1, and IL-10 genes, while inversely correlated with that of the proinflammatory TNF-α, IL-6, Keap1 and NF-κB genes expression, which agrees with other studies reporting the immunoregulatory and antioxidative abilities of SDF-1 [42]. Such contradiction was partly resolved by assuming that the role of SDF-1 in the course of DN; whether renoprotective or detrimental, is largely dictated by the levels of blood glucose [43], which was consolidated by the pattern of SDF-1 correlations observed under FBG-controlled conditions. Additionally, the nuclear receptor PPARγ; particularly the CLA-activated, is reported to interrupt the SDF-1/CXCR inflammatory signaling by abrogating the CXCR4 gene expression [35, 44], which interprets the higher improvement observed in MC-treated kidneys on the functional and structural levels. What’s more, SDF-1 reportedly co-stimulates the transcription of IL-10 crucial for the anti-inflammatory action of PPARγ [45–47], which further interprets the positive correlation found between serum SDF-1 and the renal PPARγ/IL-10 transcription.

Taken together, our data suggest that both extracts produced an antidiabetic/renoprotective effect that was overall comparable with the reference angiotensin receptor blocker; i.e. Losartan; however, with better control of the DN progression owing to their potent modulatory effect on the renal transcriptome of oxido-inflammatory mediators associated with rich content of anti-inflammatory phytoconstituents. Moreover, the biosafety of the proposed extracts enables prolonged use with no adverse reactions unlike the commonly used renoprotective xenobiotics [8].

## Conclusion

Collectively, this report presented novel information on the prophylactic capacity of two purslane seed extracts against diabetic nephropathy. Both extracts exhibited a hypoglycemic and hypolipidemic-based mechanism of renoprotection; however, through different signaling pathways that comply with the phytochemical profile of each. The phenol-rich methanol extract modulates the Keap1/Nrf2/OH-1 antioxidative signaling pathway, while the fixed oil upregulated a direct SDF-1/IL-10/PPARγ-mediated anti-inflammatory signaling pathway; both ended up suppressing the NF-κB downstream inflammatory cascade. Our findings might represent a significant pillar in the pharmaceutical industry, particularly regarding the prophylaxis and/or management of diabetes-related kidney disorders. As functional food ingredients, both purslane seed extracts showed overall biosafety besides their remarkable modulatory effect on the oxido-inflammatory axis which could be useful for various prophylactic/therapeutic purposes.

### Electronic supplementary material

Below is the link to the electronic supplementary material.

Supplementary Material 1

## Data Availability

No datasets were generated or analysed during the current study.

## Abbreviations

ACE: Angiotensin-converting enzyme
γ-GCS: γ-glutamylcysteine synthetase
ARBs: Angiotensin II-receptor blockers
CLA: Conjugated linoleic acid
DKD: Diabetic kidney disease
DM: Diabetes mellitus
DN: Diabetic nephropathy
ECM: Extracellular matrix
eGFR: estimated glomerular filtration rate
ESRD: End-stage renal disease
FBG: Fasting blood glucose
FFA: Free fatty acids
GCLC: Glutamyl cysteine synthetase
HO-1: Heme oxygenase-1
hs-CRP: High sensitivity C-reactive protein
IL: Interleukin
Keap1: Kelch-like ECH-associated protein
MAPK: Mitogen-activated protein kinase
MC: Methylene chloride extract
MO: Methanol extract
NF-κB: Nuclear factor kappa
Nrf2: Nuclear factor-erythroid-2-related factor 2
PKC: Protein kinase C
PPARγ: Peroxisome-proliferator activated receptor-gamma
ROS: Reactive oxygen radicals
SD: Sprague Dawley
SOD: Superoxide dismutase
STZ: Streptozotocin
TGF-β: Transforming growth factor-β
TNF-α: Tumor necrosis factor

## References

[1] World Health Organization. WHO fact sheet: Diabetes. 2023. https://www.who.int/news-room/fact-sheets/detail/diabetes [Accessed 31 January 2024].

[2] Zenoaga-Barbăroșie C, Berca L, Vassu-Dimov T, Toma M, Nica MI, Alexiu-Toma OA, Ciornei C, Albu A, Nica S, Nistor C, Nica R (2023). The predisposition for type 2 diabetes Mellitus and metabolic syndrome. Balkan J Med Genet.

[3] Wojcik M, Krawczyk M, Zieleniak A, Mac Marcjanek K, Wozniak LA. Chapter 14–Associations of high blood sugar with oxidative stress and inflammation in patients with type 2 diabetes. In: Harry G. Preuss, Debasis Bagchi, editors. Dietary Sugar, Salt and Fat in Human Health.2020, pp. 305–323. 10.1016/B978-0-12-816918-6.00014-7.

[4] Pan D, Xu L, Guo M (2022). The role of protein kinase C in diabetic microvascular complications. Front Endocrinol (Lausanne).

[5] Zhao L, Zou Y, Liu F (2020). Transforming growth Factor-Beta1 in Diabetic kidney disease. Front Cell Dev Biol.

[6] Wu T, Ding L, Andoh V, Zhang J, Chen L (2023). The mechanism of Hyperglycemia-Induced Renal Cell Injury in Diabetic Nephropathy Disease: an update. Life (Basel).

[7] Mitrofanova A, Merscher S, Fornoni A (2023). Kidney lipid dysmetabolism and lipid droplet accumulation in chronic kidney disease. Nat Rev Nephrol.

[8] National Kidney Foundation. Angiotensin-converting enzyme (ACE) inhibitors & angiotensin receptor blockers (ARBs). https://www.kidney.org/atoz/content/angiotensin-converting-enzyme-ace-inhibitors-angiotensin-receptor-blockers-arbs [Accessed 31 January 2024].

[9] Al-Tamimi JZ, AlFaris NA, Al-Farga AM, Alshammari GM, BinMowyna MN, Yahya MA (2021). Curcumin reverses diabetic nephropathy in streptozotocin-induced diabetes in rats by inhibition of PKCβ/p^66^Shc axis and activation of FOXO-3a. J Nutr Biochem.

[10] Ding X, Zhao H, Qiao C (2022). Icariin protects podocytes from NLRP3 activation by Sesn2-induced mitophagy through the Keap1-Nrf2/HO-1 axis in diabetic nephropathy. Phytomedicine.

[11] Putra IMWA, Fakhrudin N, Nurrochmad A, Wahyuono S (2023). A review of Medicinal plants with renoprotective activity in Diabetic Nephropathy Animal models. Life (Basel).

[12] Jin Q, Liu T, Qiao Y, Liu D, Yang L, Mao H, Ma F, Wang Y, Peng L, Zhan Y (2023). Oxidative stress and inflammation in diabetic nephropathy: role of polyphenols. Front Immunol.

[13] Hashemi M, Zandieh MA, Ziaolhagh S, Mojtabavi S, Sadi FH, Koohpar ZK (2023). Nrf2 signaling in diabetic nephropathy, cardiomyopathy and neuropathy: therapeutic targeting, challenges and future prospective. Biochim Biophys Acta Mol Basis Dis.

[14] Chauhan W, Zennadi R (2023). Keap1-Nrf2 heterodimer: a therapeutic target to Ameliorate Sickle Cell Disease. Antioxid (Basel).

[15] Chen X, Qi J, Wu Q, Jiang H, Wang J, Chen W, Mao A, Zhu M (2020). High glucose inhibits vascular endothelial Keap1/Nrf2/ARE signal pathway via downregulation of monomethyltransferase SET8 expression. Acta Biochim Biophys Sin (Shanghai).

[16] Enayati A, Ghojoghnejad M, Roufogalis BD, Maollem SA, Sahebkar A. Impact of Phytochemicals on PPAR Receptors: Implications for Disease Treatments. PPAR Res. 2022; 2022:4714914. 10.1155/2022/4714914. 10.1155/2022/4714914 PMC9453090 36092543

[17] Gao J, Gu Z (2022). The role of peroxisome proliferator-activated receptors in kidney diseases. Front Pharmacol.

[18] Uddin MK, Juraimi AS, Hossain MS, Nahar MA, Ali ME, Rahman MM. Purslane weed (Portulaca oleracea): a prospective plant source of nutrition, omega-3 fatty acid, and antioxidant attributes. ScientificWorldJournal. 2014;2014(951019). 10.1155/2014/951019. 10.1155/2014/951019 PMC3934766 24683365

[19] Osman SM, Hussein MA (2015). Purslane seeds fixed oil as a functional food in treatment of obesity induced by high fat diet in obese diabetic mice. Nutr Food Sci.

[20] Nazeam JA, El-Hefnawy HM, Omran G, Singab AN (2018). Chemical profile and antihyperlipidemic effect of *Portulaca oleracea* L. seeds in streptozotocin-induced diabetic rats. Nat Prod Res.

[21] Timoneda A, Feng T, Sheehan H, Walker-Hale N, Pucker B, Lopez-Nieves S, Guo R, Brockington S (2019). The evolution of betalain biosynthesis in Caryophyllales. New Phytol.

[22] Ahmed SA, Shaker SE, Shawky H (2022). Solvent polarity dictates the anti-inflammatory potency and mechanism of two purslane (*Portulaca oleracea*) seed extracts. J Food Biochem.

[23] Lorke D (1983). A new approach to practical acute toxicity testing. Arch Toxicol.

[24] Wang-Fischer Y, Garyantes T (2018). Improving the reliability and utility of Streptozotocin-Induced Rat Diabetic Model. J Diabetes Res.

[25] Dirnena-Fusini I, Åm MK, Fougner AL, Carlsen SM, Christiansen SC (2018). Intraperitoneal, subcutaneous and intravenous glucagon delivery and subsequent glucose response in rats: a randomized controlled crossover trial. BMJ Open Diabetes Res Care.

[26] Mohammadi Y, Zangooei M, Salmani F, Farimani AR (2023). Effect of crocin and losartan on biochemical parameters and genes expression of FRMD3 and BMP7 in diabetic rats. Turk J Med Sci.

[27] Leão T, Siqueira M, Marcondes S, Franco-Belussi L, De Oliveira C, Fernandes CE (2021). Comparative liver morphology associated with the hepatosomatic index in five neotropical anuran species. Anat Rec (Hoboken).

[28] Besseling PJ, Pieters TT, Nguyen ITN, de Bree PM, Willekes N, Dijk AH, Bovée DM, Hoorn EJ, Rookmaaker MB, Gerritsen KG, Verhaar MC, Gremmels H, Joles JA (2021). A plasma creatinine- and urea-based equation to estimate glomerular filtration rate in rats. Am J Physiol Ren Physiol.

[29] Suvarna K, Layton C. The gross room/surgical cut-up. In: Suvarna SK, Layton C, Bancroft JD, editors. Bancroft’s Theory and Practice of Histological Techniques (7th Edition). Oxford: Churchill Livingstone. 2013, pp. 95–103.

[30] Jalali J, Ghasemzadeh Rahbardar M (2022). Ameliorative effects of *Portulaca oleracea L*. (purslane) on the metabolic syndrome: a review. J Ethnopharmacol.

[31] Alemán CL, Más RM, Rodeiro I, Noa M, Hernández C, Menéndez R, Gámez R (1998). Reference database of the main physiological parameters in Sprague-Dawley rats from 6 to 32 months. Lab Anim.

[32] Ahmed SA, Aziz WM, Shaker SE, Fayed DB, Shawky H (2022). Urinary transferrin and proinflammatory markers predict the earliest diabetic nephropathy onset. Biomarkers.

[33] Eleazu CO, Iroaganachi M, Eleazu KC (2013). Ameliorative potentials of cocoyam (Colocasia esculenta L.) and unripe plantain (Musa Paradisiaca L.) on the relative tissue weights of streptozotocin-induced diabetic rats. J Diabetes Res.

[34] Costa VA, Vianna LM (2008). Biological response of spontaneously hypertensive rats to the streptozotocin administration. Braz Arch Biol Technol.

[35] Baird L, Yamamoto M (2020). The Molecular mechanisms regulating the KEAP1-NRF2 pathway. Mol Cell Biol.

[36] Scirpo R, Fiorotto R, Villani A, Amenduni M, Spirli C, Strazzabosco M (2015). Stimulation of nuclear receptor peroxisome proliferator-activated receptor-γ limits NF-κB-dependent inflammation in mouse cystic fibrosis biliary epithelium. Hepatology.

[37] Ibitoye OB, Ajiboye TO (2020). Protocatechuic acid protects against menadione-induced liver damage by up-regulating nuclear erythroid-related factor 2. Drug Chem Toxicol.

[38] Putera HD, Doewes RI, Shalaby MN, Ramírez-Coronel AA, Clayton ZS, Abdelbasset WK (2023). The effect of conjugated linoleic acids on inflammation, oxidative stress, body composition and physical performance: a comprehensive review of putative molecular mechanisms. Nutr Metab (Lond).

[39] Yang CC, Wu CH, Lin TC, Cheng YN, Chang CS, Lee KT, Tsai PJ, Tsai YS (2021). Inhibitory effect of PPARγ on NLRP3 inflammasome activation. Theranostics.

[40] Gao W, Guo L, Yang Y, Wang Y, Xia S, Gong H, Zhang BK, Yan M (2022). Dissecting the Crosstalk between Nrf2 and NF-κB response pathways in Drug-Induced toxicity. Front Cell Dev Biol.

[41] Song A, Jiang A, Xiong W, Zhang C (2021). The role of CXCL12 in kidney diseases: a friend or foe?. Kidney Dis (Basel).

[42] Jin W, Zhao Y, Hu Y, Yu D, Li X, Qin Y, Kong D, Wang H. Stromal Cell-Derived Factor-1 Enhances the Therapeutic Effects of Human Endometrial Regenerative Cells in a Mouse Sepsis Model. Stem Cells Int. 2020; 2020:4820543. 10.1155/2020/4820543 PMC7103048 32256608

[43] Shawky H, Fayed DB (2023). Assessment of circulating stromal cell-derived factor (SDF)-1 as prognostic marker of Diabetes-Induced Tubular Atrophy. Egypt J Chem.

[44] Revskij D, Haubold S, Plinski C, Viergutz T, Tuchscherer A, Kröger-Koch C, Albrecht E, Günther J, Tröscher A, Hammon HM, Schuberth HJ, Mielenz M (2022). Cellular detection of the chemokine receptor CXCR4 in bovine mammary glands and its distribution and regulation on bovine leukocytes. J Dairy Sci.

[45] Kim SR, Lee KS, Park HS, Park SJ, Min KH, Jin SM, Lee YC (2005). Involvement of IL-10 in peroxisome proliferator-activated receptor gamma-mediated anti-inflammatory response in asthma. Mol Pharmacol.

[46] Kremer KN, Kumar A, Hedin KE (2007). Haplotype-independent costimulation of IL-10 secretion by SDF-1/CXCL12 proceeds via AP-1 binding to the human IL-10 promoter. J Immunol.

[47] Li X, Lan X, Zhao Y, Wang G, Shi G, Li H, Hu Y, Xu X, Zhang B, Ye K, Gu X, Du C, Wang H (2019). SDF-1/CXCR4 axis enhances the immunomodulation of human endometrial regenerative cells in alleviating experimental colitis. Stem Cell Res Ther.

