# Supplementary material for: Portulaca oleracea L seed extracts counteract diabetic nephropathy through SDF-1/IL10/PPARγ–mediated tuning of keap1/Nrf2 and NF-κB transcription in Sprague Dawley rats
Source: Diabetol Metab Syndr. 2024 May 30;16:119. doi: 10.1186/s13098-024-01330-y (PMC11138090; doi:10.1186/s13098-024-01330-y)
Supplement: Supplementary file 1 — Supplementary Material 1 [file 13098_2024_1330_MOESM1_ESM.docx]

***Portulaca oleracea* L Seed Extracts Counteract Diabetic Nephropathy through SDF-1/IL10/PPARγ–Mediated Tuning of Keap1/Nrf2 and NF-κB Transcription in Sprague Dawley Rat Model**

**Wessam M. Aziz[
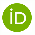
](https://orcid.org/0000-0002-3655-8490), Samia A. Ahmed[
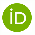
](https://orcid.org/0000-0002-5292-4040), Sylvia E. Shaker[
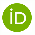
](https://orcid.org/0000-0003-1672-7231), Dalia B. Fayed[
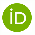
](https://orcid.org/0000-0001-5238-7516), Nadia S. Metwally[
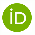
](https://orcid.org/0000-0002-3591-7148), Heba Shawky*[
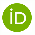
](https://orcid.org/0000-0001-8403-1143)**

Table of Contents

[**SUPPLEMENTARY FIGURES**](#Supplementary_Methods) **……...**……………………………………………………………...2

[**SUPPLEMENTARY TABLES**](#Supplementary_Tables)

[Supplementary Table 1: Primer sets used for gene expression profiling](#Primers)……………...……………....3

[Supplementary Table 2: Effect of repeated dosing of purslane extracts on body weight](#Body_weight)……………....4

[Supplementary Table 3: Effect of repeated dosing of purslane extracts on organ indices](#Organ_index)……………..5

[Supplementary Table 4: Effect of repeated dosing of purslane extracts on the blood biochemistry](#Biochemistry)…..6

[Supplementary Table 5: Correlations between hyperglycemia, oxidative stress, and the transcription levels of inflammatory cytokines in diabetic kidneys](#Correlation_matrix)………………………………………………..…7

[**SUPPLEMENTARY REFERENCES**](#Supplementary_Reference)………………………………………………………………..8


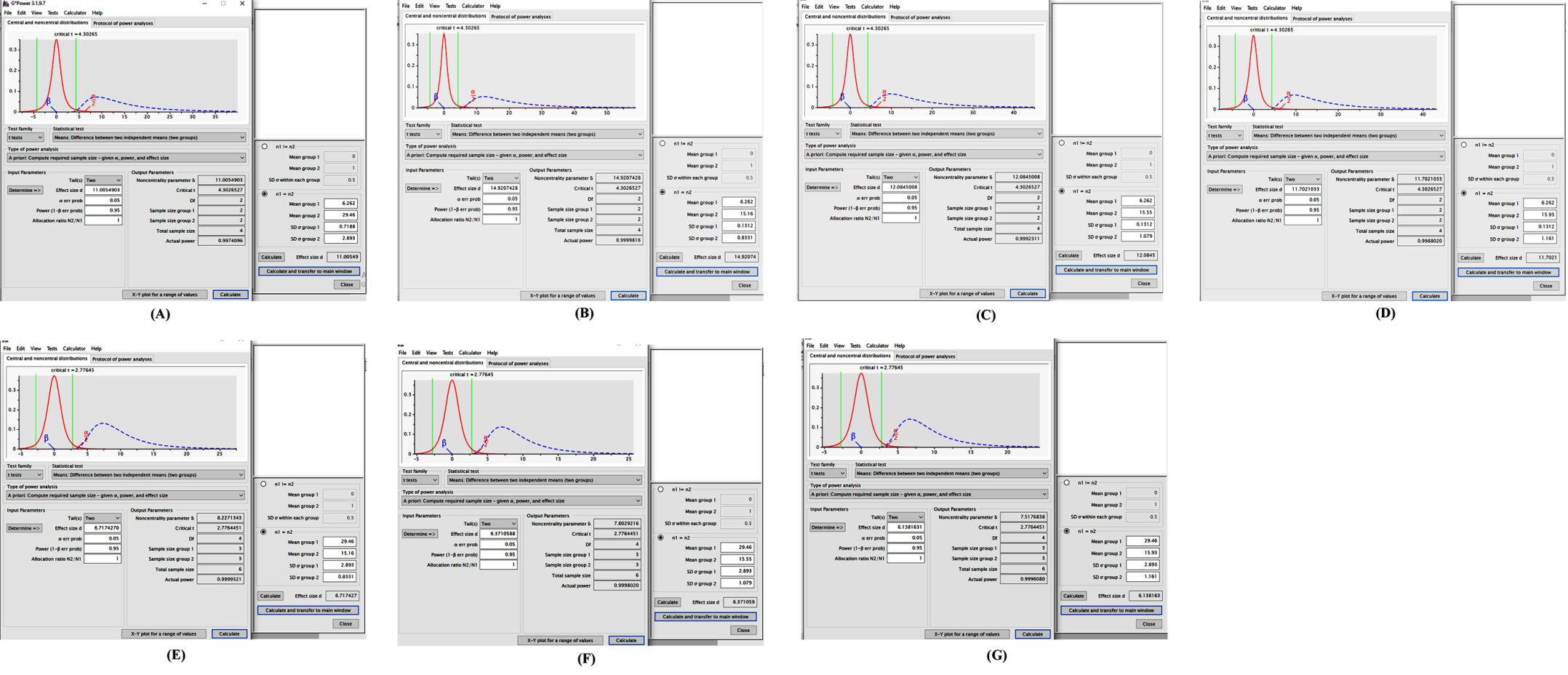
**Fig. (S1):** Determination of sample size using G-Power software. The Priori test analysis of the five independent groups included in the study revealed ~ 11 fold difference of the FBG levels in the DN group relative to sham **(A)**, while it showed ~12.902 and 6.408 fold difference in treated groups relative to sham **(B-D)** and DN **(E-G)** control groups; respectively, with mean difference of 9.655 and an average sample size required of three rats per group.

**SUPPLEMENTARY TABLES**

**Supplementary Table 1: Primer sets used for gene expression profiling**

| Gene | Primer sequence | Reference | *Primer Efficiency |
| --- | --- | --- | --- |
| *NF-κB* | **F:**5’-CGCAAAAGGACCTACGAGAC-3’  **R:** 5’-TGGGGGAAAACTCATCAAAG-3’ | **[1]** | 107.126% |
| *IL-6* | **F:**5’-TCCTACCCCACCTTCCAATGCAAC-3’  **R:** 5’-TTGGATGGTCTTGGTCCTTAGCC-3’ | **[2]** | 93.32% |
| *TNF-α* | **F:**5’-AAATGGGCTCCCTCTCATCAGTTC-3’  **R:** 5’-TCTGCTTGGTGGTTTGCTACGAC-3’ |  | 96.38% |
| *IL-10* | **F:**5’-TAACTGCACCCACTTCCCAG-3’  **R:** 5’-AGGGTCTCCTCTTCTGGCTAAC-3’ |  | 112.33% |
| *Nrf2* | **F:**5’-ACGGTGGAGTTCAATGAC-3’  **R:** 5’-GAAGAATGTGTTGGCTGTG-3’ | **[3]** | 92.75% |
| *Keap1* | **F:**5’-CTGCATCCACCACAGCAGCGT-3’  **R:** 5’-GTGCAGCACACAGACCCCGGC-3’ |  | 91.25% |
| *HO-1* | **F:**5’-GCCTGCTAGCCTGGTTCAAG-3’  **R:** 5’-AGCGGTGTCTGGGATGAACTA-3’ |  | 102.3% |
| *PPAR-γ* | **F:**5’-GGGACGCTGAAGAAGAGACCTG-3’  **R:** 5’-CACAGTCCGGTCAGAAAGTGA-3’ |  | 97.24% |
| *β-actin* | **F:** 5’‑ATCTGGCACCACACCTTC-3’  **R:** 5’-AGCCAGGTCCAGACGCA-3’ | **[1]** | - |

*Primer efficiency was calculated based on the slope of the standard curve established between Ct and log DNA copy number (amplification factor=2) using the calculator of **ThermoFisher Scientific** available from <https://www.thermofisher.com/eg/en/home/brands/thermo-scientific/molecular-biology/molecular-biology-learning-center/molecular-biology-resource-library/thermo-scientific-web-tools/qpcr-efficiency-calculator.html>

**Supplementary Table 2: Effect of repeated dosing of purslane extracts on body weight (*n*=10/group)**

| Group | Mean Body Weight (g) | | | | | |
| --- | --- | --- | --- | --- | --- | --- |
|  | **Days** | | | | | |
|  | **7** | **14** | **21** | **28** | **35** | **42** |
| Sham | 144.2 ± 2.08 | 157.6 ± 3.51 | 175 ± 1.53 | 183 ± 2.77 | 222.2 ± 3.35 | 274.2 ± 2.33 |
| MO | 144.6 ± 2.52 | 159.6 ± 2.65 | 173.4 ± 2.39 | 181 ± 2.55 | 219.4 ± 3.54 | 272.2 ± 3.22 |
| MC | 145 ± 2.08 | 155.8 ± 3.06 | 170.4± 2.88^a^ | 180.2 ± 1.8^a^ | 217 ± 2.87^a^ | 270.4 ± 2.7^a^ |

All data are expressed as mean ± SD

^a^Significant compared to the sham group in the same time point

^b^Significant compared to the MO-treated group in the same time point

**Supplementary Table 3: Effect of repeated dosing of purslane extracts on organ indices (*n*=10/group)**

| Group | Brain |  | Lungs |  | Heart |  | Liver | Kidney | Spleen | Testes |
| --- | --- | --- | --- | --- | --- | --- | --- | --- | --- | --- |
| Sham | 0.569 ± 0.02 |  | 0.565 ± 0.05 |  | 0.35 ± 0.05 |  | 4.22 ± 0.054 | 0.248 ± 0.03 | 0.456 ± 0.07 | 1.152 ± 0.07 |
| MO | 0.57 ± 0.04 |  | 0.544 ± 0.06 |  | 0.344 ± 0.03 |  | 4.198 ± 0.028 | 0.249 ± 0.04 | 0.44 ± 0.04 | 1.148 ± 0.08 |
| MC | 0.55 ± 0.03 |  | 0.562 ± 0.05 |  | 0.367 ± 0.04 |  | 4.204 ± 0.11 | 0.247± 0.04 | 0.459 ± 0.05 | 1.161 ± 0.06 |

All data are expressed as mean ± SD

*Significant compared to the corresponding organ in the sham group

**Supplementary Table 4: Effect of repeated dosing of purslane extracts on the blood biochemistry**

| Parameter | Group | | |
| --- | --- | --- | --- |
|  | **Sham** | **MO** | **MC** |
| Glucose (mmol/L) | 5.66 ± 0.47 | 4.94 ± 0.41^a^ | 5.46 ± 0.35 |
| Total Cholesterol (mg/dL) | 95.4 ± 1.95 | 81 ± 1.51^a^ | 88 ± 1.71^ab^ |
| Triglycerides (mg/dL) | 45 ± 2.59 | 33.81 ± 0.65^a^ | 28.18 ± 0.36^ab^ |
| ALT (IU/L) | 41.3 ± 1.7 | 33.7 ± 1.81^a^ | 36.2 ± 2.22^a^ |
| AST (IU/L) | 91.6 ± 4.23 | 88 ± 2.33 | 89 ± 1.52 |
| Uric Acid (mg/dL) | 1.87 ± 0.06 | 1.66 ± 0.04^a^ | 1.72 ± 0.06^a^ |
| ALB (g/dL) | 3.15 ± 0.11 | 3.2 ± 0.09 | 3.5 ± 0.22^a^ |
| BUN (mg/dL) | 14.28 ± 1.65 | 12.8 ± 0.38 | 13.66 ± 0.6 |
| Serum Creatinine (mg/dL) | 0.49 ± 0.021 | 0.59 ± 0.024^a^ | 0.56 ± 0.03^ab^ |
| Urine Creatinine (mg/dL) | 53.23 ± 0.62 | 47.65 ± 0.811^a^ | 48.68 ± 0.88^a^ |
| Cr Cl (mL/min) | 0.98 ± 0.08 | 0.88 ± 0.045 | 0.94 ± 0.06 |
| Proteinuria (mg/dL) | 11.16 ± 0.23 | 11.45 ± 0.5 | 11.01 ± 0.34 |
| Urine Volume (mL) | 13.2 ± 1.3 | 16 ± 1.41^a^ | 15.6 ± 0.89^a^ |
| eGFR (mL/min) | 1.36 ± 0.06 | 1.46 ± 0.1 | 1.36 ± 0.09 |

**Abbreviations**: **ALT**: alanine transaminase; **AST**: aspartate aminotransferase; **ALB**: albumin; **BUN**: blood urea nitrogen; **Cr Cl**: creatinine clearance, **eGFR**: estimated glomerular filtration rate

All data represent the difference between means ± SD

^a^Significant compared to the sham group

^b^Significant compared to the MO-treated group

**Supplementary Table 5: Correlations between hyperglycemia, oxidative stress, and the transcription levels of inflammatory cytokines in diabetic kidneys**

|  | Nrf2 | Keap1 | HO-1 | NF-κB | PPAR-γ | TNF-α | IL-6 | IL-10 |
| --- | --- | --- | --- | --- | --- | --- | --- | --- |
| FBG | r= -0.8621  *P*< 0.0001 | r= 0.7667  *P*= 0.0003 | r= -0.7444  *P*= 0.0005 | r= 0.9756  *P*< 0.0001 | r= -0.7443  *P*= 0.0005 | r= 0.9943  *P*< 0.0001 | r= 0.961  *P*< 0.0001 | r= -0.5427  *P*= 0.0149 |
| ROS | r= -0.8825  *P*< 0.0001 | r= 0.7395  *P*= 0.0005 | r= -0.7733  *P*= 0.0002 | r= 0.9358  *P*< 0.0001 | r= -0.6787  *P*= 0.0019 | r= 0.9688  *P*< 0.0001 | r= 0.94  *P*< 0.0001 | r= -0.4532  *P*= 0.039 |
| S/SDF-1 | r= 0.7491  *P*= 0.0025 | r= -0.8265  *P*= 0.0005 | r= 0.7138  *P*= 0.0046 | r= -0.8322  *P*= 0.0004 | r= 0.7068  *P*= 0.0051 | r= -0.8501  *P*= 0.0002 | r= -0.8329  *P*= 0.0004 | r= 0.6080  *P*= 0.0180 |
| Nrf2 |  | r= -0.8886  *P*< 0.0001 | r= 0.9635  *P*< 0.0001 | r= -0.8424  *P*< 0.0001 | r= 0.6235  *P*= 0.0049 | r= -0.8768  *P*< 0.0001 | r= -0.9272  *P*< 0.0001 | r= 0.4417  *P*= 0.0434 |
| Keap1 |  |  | r= -0.9117  *P*< 0.0001 | r= 0.762  *P*= 0.0003 | r= -0.6122  *P*= 0.0059 | r= 0.7717  *P*= 0.0002 | r= 0.8793  *P*< 0.0001 | r= -0.5001  *P*= 0.0243 |
| HO-1 |  |  |  | r= -0.7231  *P*= 0.0008 | r= 0.5102  *P*= 0.0217 | r= -0.7623  *P*= 0.0003 | r= -0.8467  *P*< 0.0001 | NS* |
| NF-κB |  |  |  |  | r= -0.8688  *P*< 0.0001 | r= 0.9858  *P*< 0.0001 | r= 0.9479  *P*< 0.0001 | r= -0.7019  *P*= 0.0012 |
| PPAR-γ |  |  |  |  |  | r= -0.7821  *P*= 0.0002 | r= -0.7449  *P*= 0.0005 | r= 0.9571  *P*< 0.0001 |
| TNF-α |  |  |  |  |  |  | r= 0.8889  *P*< 0.0001 | r= -0.8644  *P*< 0.0001 |
| IL-6 |  |  |  |  |  |  |  | r= -0.8035  *P*<0.0001 |

***NS**: non-significant

**SUPPLEMENTARY REFERENCES**

1. Miri S, Rasooli A, Brar SK. Data on changes of NF-κB gene expression in liver and lungs as a biomarker and hepatic injury in CLP-induced septic rats. Data Brief. 2019; 25:104117. <https://doi.org/10.1016/j.dib.2019.104117>
2. Hammad AM, Ibrahim YA, Khdair SI, Hall FS, Alfaraj M, Jarrar Y, Abed AF. Metformin reduces oxandrolone- induced depression-like behavior in rats via modulating the expression of IL-1β, IL-6, IL-10 and TNF-α. Behav Brain Res. 2021; 414:113475. [https://doi.org/10.1016/ j.bbr.2021.113475](https://doi.org/10.1016/%20j.bbr.2021.113475)
3. Nakai K, Fujii H, Kono K, Goto S, Kitazawa R, Kitazawa S, Hirata M, Shinohara M, Fukagawa M, Nishi S. Vitamin D activates the Nrf2-Keap1 antioxidant pathway and ameliorates nephropathy in diabetic rats. Am J Hypertens. 2014; 27(4):586-595. <https://doi.org/10.1093/ajh/hpt160>
